# CD38^hi^ macrophages promote fibrotic transition following acute kidney injury by modulating NAD^+^ metabolism

**DOI:** 10.1016/j.ymthe.2025.04.039

**Published:** 2025-05-09

**Authors:** Weijian Yao, Menghan Liu, Zehua Li, Lei Qu, Shuang Sui, Chengang Xiang, Lei Jiang, Suxia Wang, Gang Liu, Ying Chen, Li Yang

**Affiliations:** 1 Renal Division, Peking University Institute of Nephrology, Key Laboratory of Renal Disease-Ministry of Health of China, Key Laboratory of Chronic Kidney Disease Prevention and Treatment (Peking University)-Ministry of Education of China, Research Units of Diagnosis and Treatment of Immune-mediated Kidney Diseases-Chinese Academy of Medical Sciences, Beijing Key Laboratory of Precision Medicine and New-drug/Equipment Development for Severe Kidney Disease, Peking University First Hospital, Beijing 100034, P.R. China; 2 Laboratory of Electron Microscopy Pathological Center, Peking University First Hospital, Beijing 100034, P.R. China

**Keywords:** acute kidney injury, renal fibrosis, scRNA sequencing, CD38hi macrophages, NAD+, senescence

## Abstract

Acute kidney injury (AKI) encompasses a spectrum of conditions, varying from mild and self-limiting to severe cases that can lead to chronic kidney disease (CKD). Macrophages are crucial in the progression from AKI to CKD, yet the diversity of macrophage subsets complicates the identification of key functional types. We established a detailed single-cell atlas of mononuclear macrophages from the onset of AKI through its progression to CKD. Our results indicate that a macrophage subset with high CD38 expression is closely linked to renal fibrosis following AKI in both mouse model and AKI patients. These CD38^hi^ macrophages, derived from resident macrophages via Csf1 signaling, secrete the NAD-depleting enzyme CD38, inducing senescence in renal tubular cells and promoting chronic inflammation and renal fibrosis. Knocking out CD38 in macrophages elevated renal NAD levels, reducing senescence and fibrotic responses. Furthermore, we initiated a dosing regimen for a CD38 inhibitor, demonstrating its potential to reduce fibrosis post-AKI, suggesting that targeting CD38^hi^ macrophages-mediated NAD^+^ metabolism could be a promising therapy to halt AKI to CKD progression.

## Graphical abstract

**Figure:**
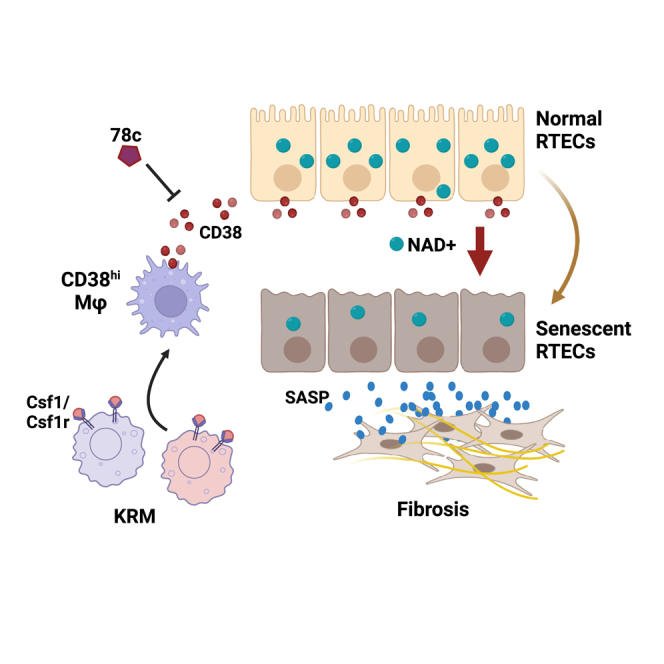

## Teaser

This study identifies CD38^hi^ macrophages as key drivers of renal fibrosis in the transition from acute to chronic kidney injury (AKI to CKD). Targeting CD38-mediated NAD metabolism offers a promising therapeutic strategy, supported by validated dosing regimens in mouse models, highlighting potential interventions for chronic AKI progression in humans.

## Introduction

Acute kidney injury (AKI) comprises a range of acute renal conditions defined by a sudden loss of excretory kidney function.^1^ Evidence shows that maladaptive tissue repair after AKI is the major underlying cause of chronic kidney disease (CKD) progression and even end stage renal disease (ESRD).^2^^,^^3^ Macrophages are key immune cells in AKI, possibly determining the fate of disease progression.^4^ Macrophages in the damaged site can either be derived from embryonic tissue-resident macrophages or be recruited from bone marrow-derived monocytes.^5^^,^^6^^,^^7^ In the local renal environment, macrophages differentiate into various phenotypes and alter the microenvironment via a paracrine effect, thus playing vital roles in mediating renal injury, inflammation, repair, and fibrosis.^8^^,^^9^ Their high plasticity means that both residential and recruited, pro-inflammatory and pro-fibrosis macrophages might coexist during AKI-CKD progression. Therefore, therapeutic strategies that deplete macrophages or transfer macrophages back into injured kidneys at different stages of AKI have produced controversial results.^10^ Thus, it is important to systematically analyze and identify the subtype classification, organ origin, and functional properties of macrophages throughout the AKI-CKD process, to better direct specific targeted therapy to macrophages.

Using single-cell RNA sequencing (scRNA-seq) technology, we previously generated a mononuclear phagocyte (MPC) atlas containing monocytes, macrophages, and dendritic cells in the acute phase (within 3 days) of ischemia reperfusion injury (IRI)-AKI. We have successfully identified a monocyte-derived Ly6c^hi^S100a8/a9^hi^ macrophage subset as the first responder to AKI, playing a pivotal role in the initiation and amplification of renal inflammation.^11^ However, during the chronic progression of AKI, either infiltrated monocytes or kidney-resident macrophages (KRMs) can be activated and differentiate to promote tissue repair or maladaptive repair until renal fibrosis.^12^ The complicated macrophage subsets and their biological functions during the progression of AKI to CKD remain unclear. In the present study, we constructed a comprehensive mononuclear macrophage atlas throughout the progression of AKI to CKD. This atlas incorporated data from the acute phase at days 1 and 3, as reported in our previous publication, and newly generated data from the repair and chronic phases at days 5, 7, 10, 17, and 28 post-IRI. The major subsets of macrophages present during the chronic phase were classified, and CD38^hi^ macrophages were suggested as a primary macrophage subset associated with fibrosis.

As a cell surface receptor, CD38 was initially considered as a surface activation marker of T cells and B cells, but was later found to be expressed in other immune cells, including macrophages.^13^^,^^14^ Notably, CD38^+^ M1 type macrophages have been implicated in infectious diseases and non-sterile inflammatory conditions. Lipopolysaccharide (LPS) exposure has been shown to upregulate CD38 expression in cultured macrophages. Furthermore, blocking CD38 with quercetin significantly alleviated kidney dysfunction and pathological changes, as well as reduced inflammatory cell accumulation, by diminishing nuclear factor κB signaling activation in a mouse model of LPS-induced AKI.^15^ In aging metabolic tissues, inflammatory cytokines secreted by senescent cells can induce macrophage proliferation and the expression of CD38, contributing to the accumulation of pro-inflammatory CD38^+^ M1-type macrophages in visceral white adipose tissue and the liver.^16^ Additionally, CD38^+^ macrophages have been associated with pro-inflammatory roles in ischemic brain injury, inflammatory bowel disease, and systemic lupus erythematosus.^17^^,^^18^^,^^19^ However, to date, there have been no reports concerning the presence of CD38^+^ macrophages in the context of IRI-induced AKI.

Therefore, this study aimed to explore the origin of CD38^hi^ macrophages following IRI-AKI and to elucidate the functional mechanisms of CD38^hi^ macrophages in the progression from AKI to CKD through macrophage-specific depletion of CD38. Moreover, we applied a CD38-specific small molecule inhibitor, 78c, in unilateral ischemia-reperfusion (uIRI) and unilateral ureteral obstruction (UUO) mouse models to investigate the therapeutic effects of CD38 inhibition on alleviating renal fibrosis subsequent to renal injury.

## Results

### Renal mononuclear macrophage atlas throughout the AKI to CKD process

To establish a renal fibrosis model, uIRI surgery was performed on the left kidneys, and the mice were sacrificed before (normal control, NC) or on days 1, 3, 5, 7, 10, 17, and 28 post-surgery (Figure S1A). The kidneys were stained using periodic acid-Schiff (PAS) and Masson staining. On day 1 after uIRI, the kidneys showed tubular epithelial cell death, interstitial inflammatory cell infiltration, and capillary congestion, representing the acute tissue injury phase of AKI (Figure S1B). On days 3–7, renal tubular epithelial cell (RTEC) division and proliferation were observed, and cell polarity was partially restored, representing the repair period (Figure S1B). From postoperative days 10 to 28, tubular epithelial cell atrophy, interstitial inflammatory cell infiltration, and fibrosis were observed, representing the chronic fibrotic stage of kidney injury (Figures S1B and S1C).

**Figure 1:**
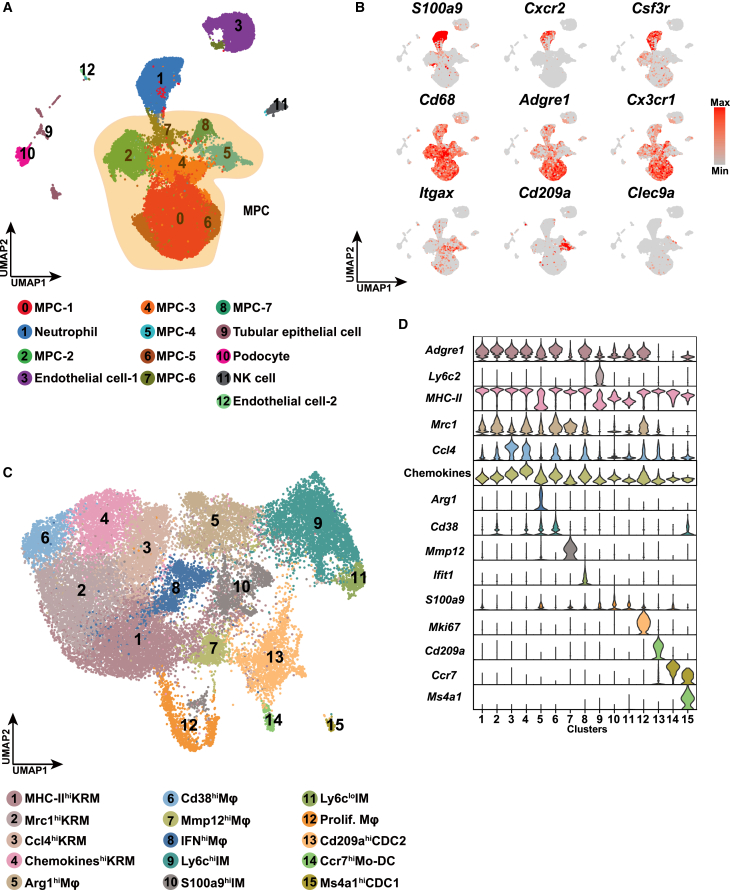
Single-cell transcriptomics profiling of kidney mononuclear phagocytes at hemostasis (NC) and different time points after uIRI (A) UMAP plot of 38,074 sorted cells that passed quality-control. Clusters 0, 2, 4, 5, 6, 7, and 8 were MPCs. (B) Feature plots of markers for neutrophils (*S100a9*, *Cxcr2*, and *Csf3r*), macrophages (*Cd68*, *Adgre1*, and *Cx3cr1*), and dendritic cells (*Itgax*, *Cd209a*, and *Clec9a*). (C) UMAP plot colored by MPC clusters depicting the MPC annotation. (D) Stacked violin plot of key genes in each MPC cluster. “Chemokines” is a curated gene set comprising *Ccl2*, *Ccl7*, *Ccl12*, *Cxcl2*, *Ccl3*, and *Ccl4*. MPC, mononuclear phagocyte; KRM, kidney resident macrophage; IM, infiltrating macrophage; hi, high; lo, low; Prolif., proliferating; Mϕ, macrophage.

Single-cell suspensions from the left kidneys on each time point were collected, CD45^+^CD11b^+^ or CD45^+^F4/80^+^ cells were sorted out, and then mixed in a 1:1 ratio for subsequent scRNA-seq (Figure S1D). Through data processing, 38,074 cells that passed quality control (Figure S1E) were divided into 13 cell clusters (C0–C12) (Figure 1A) using Harmony integration and UMAP (uniform manifold approximation and projection) plotting. The cell types were defined by the anchor gene expression in each cluster (Figures 1A and S2; Table S1). We could annotate seven mononuclear phagocyte (MPC) populations (C0, C2, C4, C5, C6, C7, and C8) containing 27,544 cells according to their representative genes (*Cd68*, *Adgre1*, *Cx3cr1*, *Itgax*, *Cd209a*, and *Clec9a*) (Figure 1B).

An MPC cell atlas was generated through unsupervised clustering of the 27,544 cells. The 15 identified MPC clusters (C1–C15) exhibited distinct marker gene expression patterns (Figures 1C and 1D; Table S2). Clusters C1–C11 were characterized by the high expression of monocyte or macrophage-specific genes such as *Ly6c2* and *Adgre1* (Figure 1D). Among these, four subsets (C1–C4) were present in the normal kidneys (Figure S3A) and displayed high expression of KRM-associated genes (*C1qa*, *C1qc*, *Ms4a7*, *Cd81*, and *Cx3cr1*), which we defined as KRM clusters: C1 (major histocompatibility complex [MHC]-II^hi^ KRM), which highly expressed MHC-II genes (*H2-Eb1*, *H2-Ab1*, *H2-DMb1*, *H2-Aa*, and *H2-DMa*); C2 (Mrc1^hi^ KRM), associated with high expressions of *Apoe*, *Fcrls*, and *Igf1*; C3 (Ccl4^hi^ KRM), which highly expressed inflammation-related genes (*Ccl4*, *Ccl3*, *Nfkbiz*, *Fos*, and *Junb)*; C4 (chemokines^hi^ KRM), expressing a range of chemokine-related genes (*Ccl7*, *Ccl12*, *Ccl2*, *Pf4*, *Ccl4*, and *Cxcl16*) (Figures S3B and S3C). In addition, four subsets (C5–C8), which emerged only after injury (Figure S3A), also expressed KRM characteristic genes and were classified as KRM-like macrophage subsets. These included C5 (Arg1^hi^ macrophages), C6 (CD38^hi^ macrophages), C7 (Mmp12^hi^ macrophages), and C8 (IFN^hi^ macrophages). Three additional subsets (C9–C11), characterized by the expressions of *Ly6c*, *S100a9*, *S100a8*, and *Nr4a1*, were defined as infiltrating macrophages (IMs): C9 (Ly6c^hi^ IM), C10 (S100a9^hi^ IM), and C11 (Ly6c^lo^ IM). Finally, C12 represented a proliferating cell cluster, while C13–C15 were identified as dendritic cell clusters (Figures 1C and 1D).

### CD38^hi^ macrophages are the key fibrosis-associated macrophage subpopulation in the chronic phase after AKI

**Figure 2:**
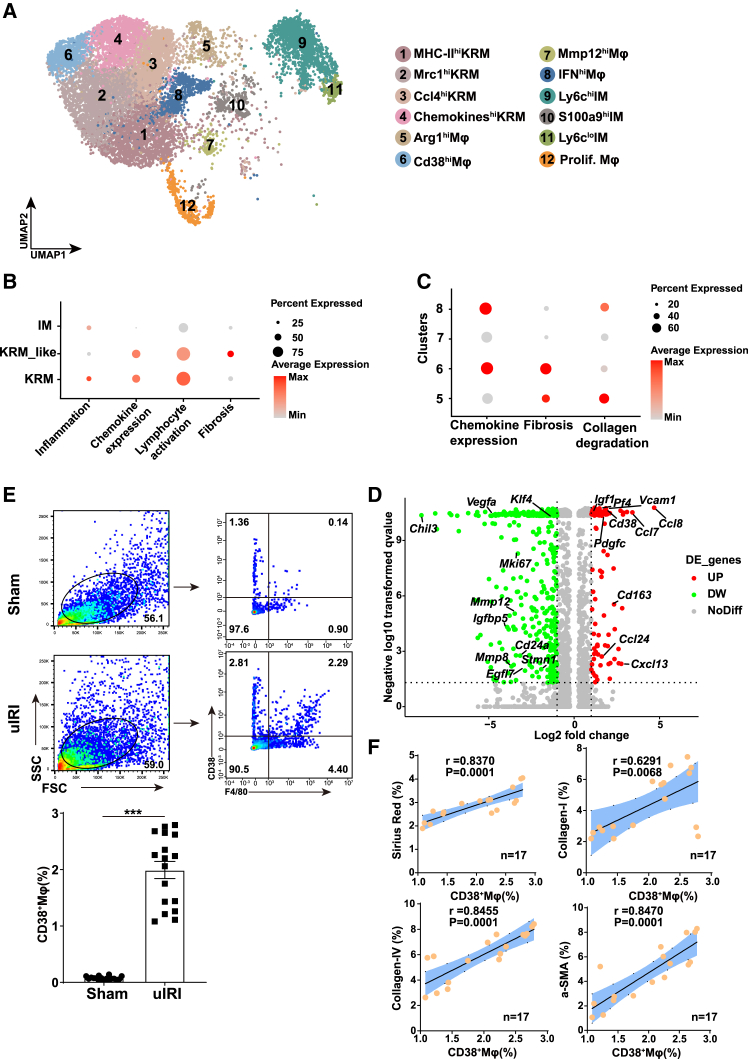
CD38^hi^ macrophages are the key fibrosis-associated macrophage subpopulation in the chronic phase after AKI (A) UMAP graph colored by MPC clusters depicting the MPC annotation in the chronic phase (day 10, day 17, and day 28) post-IRI. (B) Dot plot showing the gene set score comparison of inflammation, chemokine expression, lymphocyte activation, and fibrosis functions between kidney KRM, KRM-like macrophages emerged after injury, and IM in the chronic phase post-IRI. (C) Dot plot showing the gene set score comparison of chemokine expression, fibrosis and collagen degradation functions between the four KRM-like macrophage clusters (C5, C6, C7, and C8). (D) Volcano plot displaying the differentially expressed genes between CD38^hi^ macrophages and the other macrophage clusters. (E) Representative flow cytometry plot of CD38^hi^ macrophages gated by F4/80 and CD38 fluorescence intensity from kidney samples on day 17 post-uIRI. Bar graph statistics of the percentage of CD38^hi^ macrophages in the chronic phase (day 10, day 17, and day 28) post-uIRI; *n* = 13 for sham and *n* = 17 for uIRI; Student’s t test; ∗∗∗ *p* < 0.001. (F) Correlation analysis of the percentage of kidney CD38^hi^ macrophages and the degree of renal fibrosis in the chronic phase (day 10, day 17, and day 28) post-uIRI. *n* = 17 for uIRI samples. Pearson correlation analysis.

To investigate the major fibrosis-related macrophage subpopulation during the chronic phase following AKI, we extracted the cells from the 12 monocyte/macrophage clusters (C1–C12) identified in the MPC atlas that were present in the kidneys on days 10, 17, and 28 post-uIRI for integrative analysis (Figure 2A). Utilizing curated functional gene sets (Table S3), we assessed scores related to inflammation, chemokine expression, lymphocyte activation, and fibrosis among the KRM (C1–C4), kidney macrophages after injury (KRM-like, C5–C8), and IM (C9–C11) subtypes. Notably, the macrophages after injury (KRM-like) exhibited the highest fibrosis-related scores (Figure 2B). Among the four KRM-like clusters (C5–C8) that emerged post injury, the CD38^hi^ macrophage subset (C6) presented the highest fibrosis score and expressed several key fibrosis-related genes, including *Igf1*, *Vcam1*, *Pdgfc*, *Pf4*, and *Cd38* (Figures 2C and 2D). Flow cytometry analysis revealed a significant increase in CD38^hi^ macrophages during the chronic phase of AKI (Figure 2E). Furthermore, the percentage of CD38^hi^ macrophages in the mouse kidney during the chronic phase after uIRI correlated significantly with the degree of kidney fibrosis (Figures 2F and S3D).

To further validate the persistence of CD38^hi^ macrophages in the chronic phase of IRI-AKI, we used the scRNA-seq data from a previously published study,^20^ which analyzed sorted kidney CD45^+^ cells from repairable (uIRI 30 min with contralateral kidney nephrectomy) and irreparable (uIRI 30 min without contralateral kidney nephrectomy) mouse IRI models. Among the 18 clusters of MPCs in this re-analyzed reference dataset, a similar macrophage cluster was defined (C6) using the gene set specific to the CD38^hi^ macrophages in our MPC atlas (Figures S4A–S4C). This CD38^hi^ cluster did not exist under normal conditions, but appeared in the kidney at day 7 after injury in both IRI models. Interestingly, the predominant CD38^hi^ macrophage population was detected in the irreparable IRI mouse model at 28 days after injury (Figure S4D).

**Figure 3:**
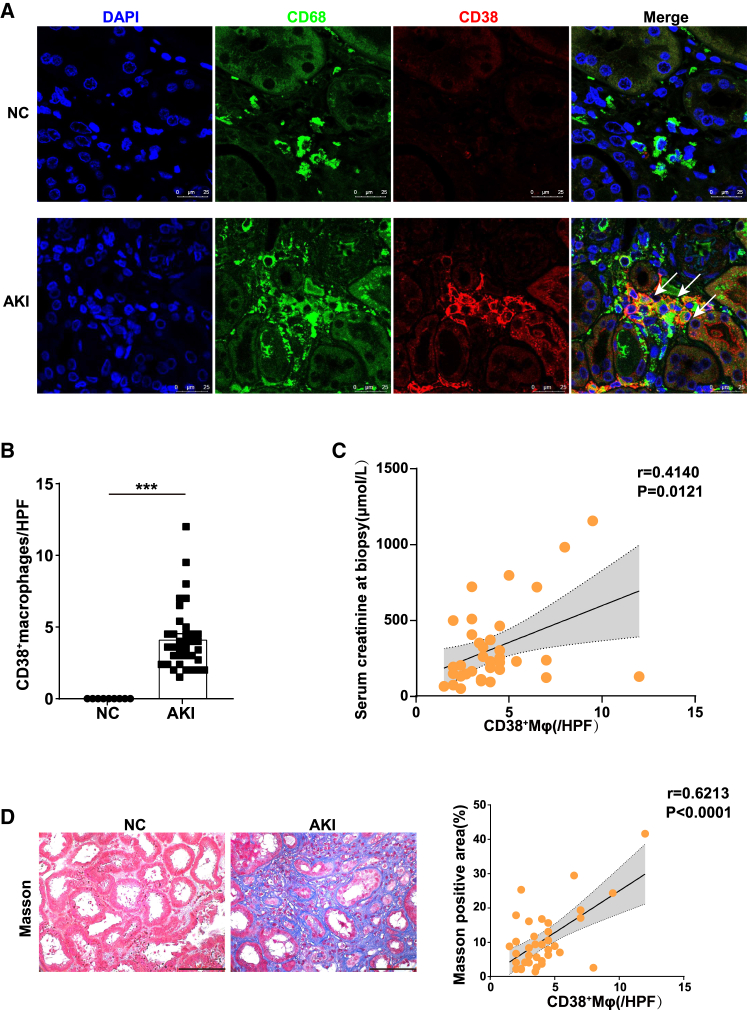
CD38^+^ macrophages in human kidneys with acute tubular injury (A) Representative images of immunofluorescence co-staining of CD68 (green) and CD38 (red) in NC and AKI kidneys. Scale bars, 50 μm. White arrows indicate cells with CD68 and CD38 co-expression. (B) Bar graph showing the averaged numbers of CD38^+^macrophages per HPF in NC and AKI kidneys. *n* = 9 for NC and *n* = 36 for AKI. Ten images were taken for each sample. Student’s t test; ∗∗∗*p* < 0.001. (C) Correlation analysis of kidney CD38^+^ macrophages and serum creatinine level at renal biopsy. Pearson correlation analysis. (D) Correlation analysis of the kidney CD38^+^ macrophages and fibrosis positive areas in AKI kidneys. *n* = 36 for AKI. Representative images of Masson staining on NC and AKI kidneys are shown on the left. Scale bars, 100 μm. Ten images were taken, and the percentage of Masson positive area was averaged for each sample. Pearson correlation analysis. NC, normal control; HPF, high-powered field.

The presence of CD38^+^ macrophages was further explored in the biopsied kidney tissues from patients with AKI using double immunofluorescence staining with anti-CD68 and anti-CD38 antibodies. CD38^+^ macrophages were absent in normal kidney tissues from paraneoplastic kidneys (Figure 3A). In contrast, CD38^+^ macrophages were detected in the renal interstitium of patients with AKI, particularly surrounding severely injured tubules (Figure 3A). The number of CD38^+^ macrophages was significantly increased in AKI patients compared to normal controls (Figure 3B). Correlation analysis revealed that the number of CD38^+^ macrophages positively correlated with serum creatinine levels at the time of renal biopsy (Figure 3C) and with the degree of renal fibrosis in these patients (Figure 3D). These findings suggest that CD38^+^ macrophages represent a key fibrosis-associated macrophage subset that persists during the chronic phase following human AKI.

### CD38^hi^ macrophages originate from activated renal resident macrophages in the acute injury phase

**Figure 4:**
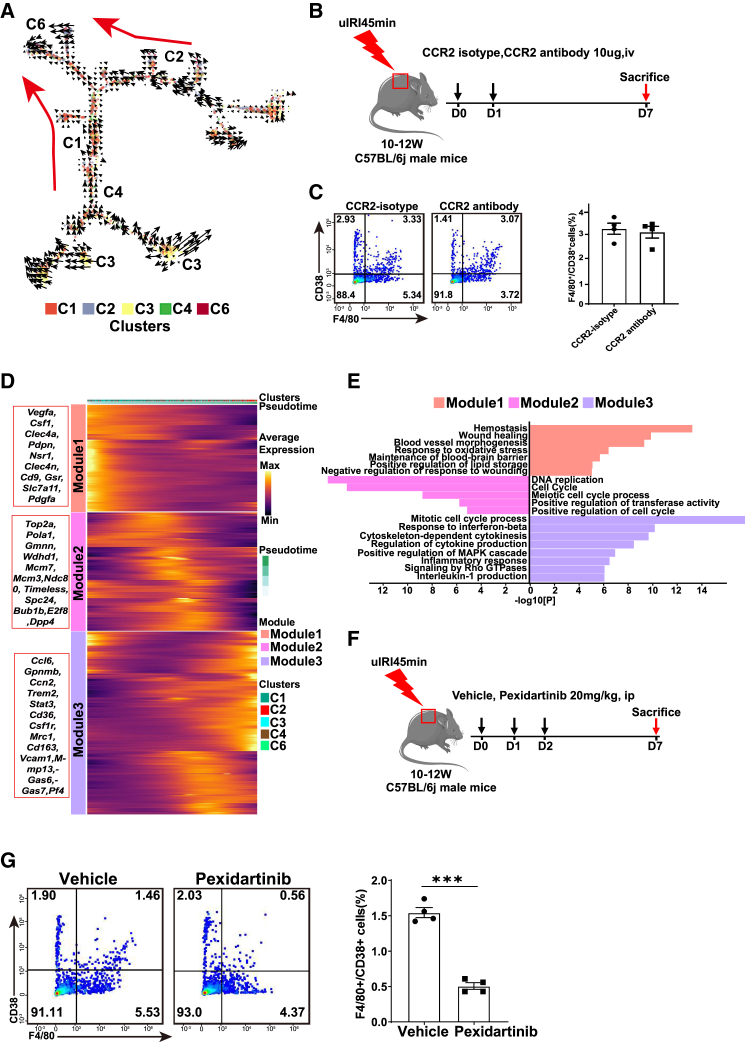
CD38^hi^macrophages originate from renal resident macrophages in the acute injury phase of AKI (A) KRM (C1–C4) and CD38^hi^macrophages (C6) developmental transition as revealed by RNA velocity. (B and C) CCR2 antibody-blocking experiment in uIRI animal model. Experimental procedures are presented by flow chart in (B); flow cytometry analysis in (C) presents the percentage of kidney CD38^hi^ macrophages at day 7 post-uIRI. *n* = 4 (D) Heatmap showing genes whose expression changed significantly along the pseudotime line in kidney macrophage clusters C1–C4 and C6, and their enrichment based on the kinetic trend of pseudo-temporal expression patterns. (E) Bar graph displaying the enriched GOBP terms of the three gene modules. (F and G) Exidartinib-treatment experiment in uIRI animal model. Experimental procedures are presented by flow chart in (F); flow cytometry analysis in (G) shows the percentage of kidney CD38^hi^ macrophages at day 7 post-uIRI. *n* = 4; Student’s t test; ∗∗∗*p* < 0.001.

Immunofluorescence staining of kidney tissues from the uIRI mouse model revealed that CD38^hi^ macrophages were largely absent in normal mouse kidney but began to emerge in the injured kidneys by day 3 post-uIRI, persisting throughout the chronic phase of AKI (Figure S5). RNA velocity analysis, conducted to identify the direction of cellular conversion between the KRM populations (C1–C4) and CD38^hi^ macrophages (C6) using dataset from normal kidneys, as well as from day 1 and day 3 post-uIRI, indicated that CD38^hi^ macrophages may originate from the C1-MHC-II^hi^KRM and C2-Mrc1^hi^KRM subsets (Figure 4A). The monocyte chemoattractant protein 1 (MCP-1 or CCL2)/CCR2 signaling axis has been proven to mediate monocytes homing to the post-ischemic kidney.^21^^,^^22^ Therefore, we employed a CCR2 neutralizing antibody to inhibit monocyte infiltration in the uIRI mouse model (Figure 4B). We detected no significant difference in the percentage of CD38^hi^ macrophages in the kidney on day 7 post-uIRI between the CCR2 neutralizing antibody-treated group and the isotype control group (Figure 4C). This finding suggests that interference with monocyte infiltration did not impact the emergence of CD38^hi^ macrophages, further supporting the notion that CD38^hi^ macrophages may originate from KRMs. In addition, we detected bromodeoxyuridine (BrdU) incorporation in CD38^hi^ macrophages from kidneys on day 7 post-uIRI, following the injection of BrdU one day prior to kidney collection. Flow cytometry was performed on single-cell suspensions from the kidneys, and staining was performed for F4/80, CD38, and either BrdU or Ki67. Approximately 30% of CD38^hi^ macrophages were found to be positive for BrdU or Ki67, indicating that these macrophages are in a state of proliferation within the post-AKI microenvironment (Figure S6A).

To further elucidate the differentiation process and potential molecular mechanisms underlying the differentiation of KRM into CD38^hi^ macrophages, we aligned dynamic genes along their developmental trajectories according to pseudotemporal changes. Three gene expression modules were identified based on the dynamic change characteristics (Figure 4D). Gene module 1 (initiation module) contained genes such as *Vegfa*, *Csf1*, *Nsr1*, and *Clec4a*, enriched for Gene Ontology Biological Process (GOBP) of hemostasis and wound healing; gene module 2 (intermediate stage) contained a large number of cell cycle-related genes, such as *Top2a*, *Dpp4*, *Mcm3*, *Mcm7*, and *Gmnn*, suggesting a cell proliferating status; gene module 3 (final stage) contained *Trem2*, *Ccl6*, *Gpnmb*, *Stat3*, *Cd36*, *Csf1r*, and was enriched for functions associated with inflammation and cell chemotaxis (Figures 4D and 4E).

In addition, through protein-protein interaction (PPI) enrichment analysis using STRING-db, we found that Stat1, Stat3, and Csf1r were key hub proteins in gene module 3 (Figure S6B). Kidney *Csf1* gene expression were increased as early as one day post-injury, peaking at days 7–17 during AKI to CKD progression (Figure S6C). Immunofluorescence staining showed that Csf1 was expressed both on the RTECs and in the interstitial cells in the injured kidneys (Figure S6D). When we used Csf1r inhibitor pexidartinib to treat the uIRI mice at postoperative days 0, 1 and 2, and subsequently collected kidneys on day 7 post-surgery (Figure 4F), we found that in the vehicle control group, the proportion of CD38^hi^macrophages was 1.46%. In contrast, the proportion of CD38^hi^macrophages in pexidartinib-treated group was significantly reduced to 0.56% (*p* < 0.05) (Figure 4G). Collectively, these data indicate that blocking Csf1/Csf1r signaling significantly decreases the number of CD38^hi^ macrophages in the kidney following IRI, supporting our hypothesis that Csf1/Csf1r-related pathways are involved in the differentiation of CD38^hi^ macrophages.

### CD38^hi^ macrophages deplete NAD by secreting CD38 and promote the senescent phenotype of RTECs

**Figure 5:**
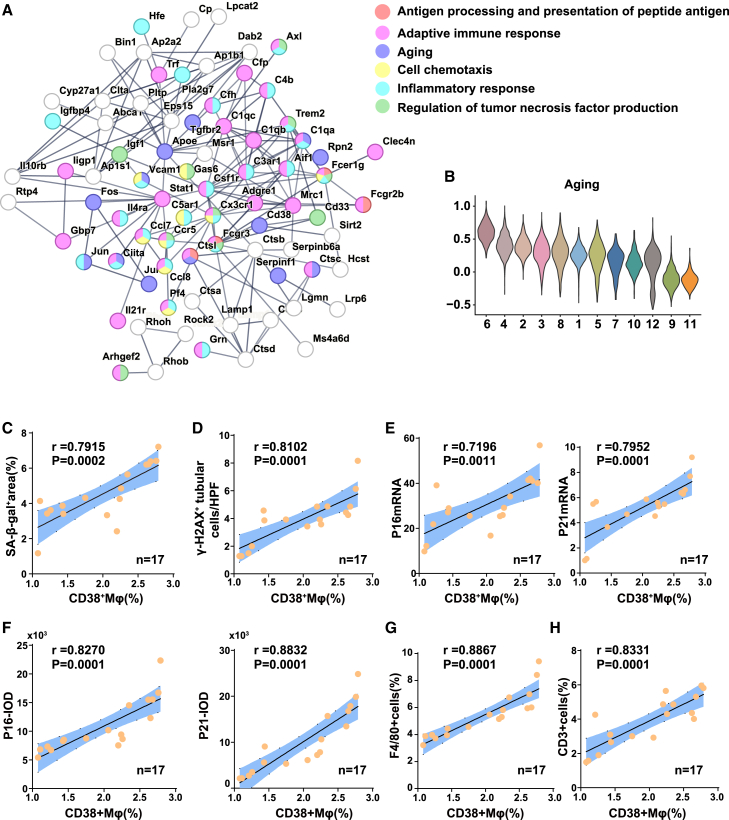
CD38^hi^ macrophages are associated with kidney cellular senescence and chronic inflammation (A) Protein-protein interaction (PPI) enrichment analysis of CD38^hi^ macrophages enriched genes in chronic phase of AKI using STRING-db (https://cn.string-db.org/). (B) Violin plot showing the score comparison of curated “aging” gene set among kidney macrophages in the chronic phase. (C–H) Correlation analysis of the percentage of kidney CD38^hi^ macrophages and SA-β-gal^+^ area, γ-H2AX^+^ tubular cells, *p16* mRNA and protein expression, *p21* mRNA and protein expression, F4/80^+^ macrophages, and CD3^+^ T cells in 17 kidney samples during the chronic phase (day 10, day 17, and day 28) post-uIRI. Original data used for performing this correlation analysis, including representative images of SA-β-gal, γ-H2AX, P16, P21, CD3 staining, flow cytometry plot of F4/80^+^ macrophages, and p16 and p21 mRNA relative expression data are presented in Figure S7.

To explore the possible roles of CD38^hi^ macrophages in AKI-CKD progression, we performed protein interaction analysis of highly expressed genes in CD38^hi^ macrophage using the Search Tool for the Retrieval of Interacting (STRING) database. In addition to common biofunctional entries associated with macrophages, such as “antigen processing and presentation of peptide antigen,” “adaptive immune response,” and “cell chemotaxis,” we identified “aging” as a distinct function significantly enriched in CD38^hi^ macrophages compared to other clusters (Figures 5A and 5B). We further examined the relationship between CD38^hi^ macrophages and markers of cellular senescence. Our analysis revealed a significant correlation between the number of CD38^hi^ macrophages in injured kidney tissues from days 1, 3, 5, 7, 10, 17, and 30 post-uIRI and the degree of senescence-associated β-galactosidase (SA-β-gal) staining (Figures 5C and S7A), the quantity of γ-H2AX + RTECs (Figures 5D and S7B), and the expression levels of p16 and p21 (Figures 5E, 5F, S7C, and S7D). Moreover, the number of CD38^hi^ macrophages correlated with the number of total F4/80^+^ macrophages and CD3^+^ T cells (Figures 5G, 5H, S7E, and S7F). These results suggest that CD38^hi^ macrophages are involved in cellular senescence and chronic inflammation following AKI.

**Figure 6:**
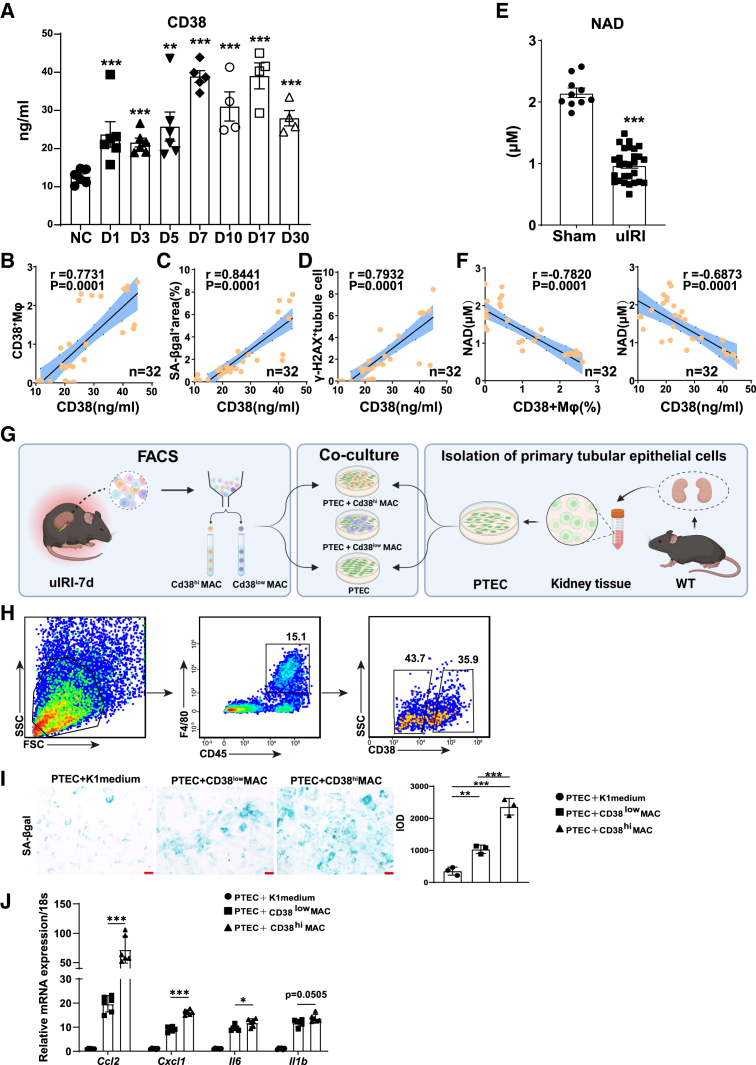
CD38^hi^ macrophages deplete renal NAD^+^ and promote the senescence of PTECs (A) Quantification of CD38 in normal control and uIRI kidneys using ELISA. Student’s t test. ∗∗*p* < 0.01 and ∗∗∗*p* < 0.001. (B–D) Correlation analysis of the kidney CD38 protein level and the percentage of CD38^hi^ macrophages (B), SA-β-gal^+^ area (C), and γ-H2AX^+^ tubular cells (D) from kidney samples on days 1, 3, 5, 7, 10, 17, and 30 post-uIRI. *n* = 32 for uIRI kidney samples. (E) The quantification of kidney NAD level between the sham and post-uIRI groups. *n* = 10 for sham group and *n* = 28 for uIRI group; Student’s t test, ∗∗∗*p* < 0.001. (F) Correlation analysis of the kidney CD38 protein, CD38^hi^ macrophages, and renal NAD level from kidney samples on days 1, 3, 5, 7, 10, 17, and 30 post-uIRI. *n* = 32 for uIRI kidney samples. (G–J) CD38^hi^, CD38^low^macrophages, and PTEC coculture experiment. (G) Flow chart of CD38^hi^, CD38^low^ macrophage isolation, and coculture with PTECs. (H) Flow cytometry plot showing the gating strategy of isolation of CD38^hi^, CD38^low^macrophages at day 7 post-uIRI. (I) Representative images of SA-β-gal staining after coculture of CD38^hi^, CD38^low^macrophages with PTECs. Scale bars, 70 μm. *n* = 3; Student’s t test; ∗∗*p* < 0.01 and ∗∗∗*p* < 0.001. (J) Expression of SASP genes in PTECs cocultured with CD38^hi^, CD38^low^macrophages. *n* = 6; Student’s t test; ∗*p* < 0.05 and ∗∗∗*p* < 0.001. PTEC, primary cultured tubular epithelial cells.

Next, the molecular mechanism of CD38^hi^ macrophages in AKI-CKD progression was investigated. Enzyme-linked immunosorbent assay (ELISA) revealed that CD38 protein levels increased in the kidney after IRI, peaking during the chronic phase of AKI (Figure 6A). More importantly, the kidney CD38 level correlated positively with the number of CD38^hi^ macrophages (Figure 6B), the degree of SA-β-gal staining (Figure 6C), and the number of γ-H2AX + RTECs (Figure 6D) in the injured kidney. Given that CD38 functions as a significant NAD^+^-depleting enzyme, we proceeded to assess the levels of NAD^+^ in the kidney. The NAD^+^ level was significantly decreased (by approximately 50%) in the chronically injured kidney compared to the sham-operated controls (Figure 6E), and both the number of CD38^hi^ macrophages and the CD38 protein level correlated negatively with renal NAD^+^ levels (Figure 6F).

To test the direct effect of CD38^hi^ macrophages on renal tubular cells, we isolated CD38^hi^ macrophages from uIRI kidneys on day 7 post-injury. These isolated macrophages were then employed to treat primary cultured mouse renal tubular cells (PTECs) in a 1:100 seeding ratio (Figures 6G and 6H). Compared to PTECs treated with CD38^low^macrophages, those cocultured with CD38^hi^ macrophages exhibited elevated SA-β-gal expression (Figure 6I). Additionally, we assessed the expression of cytokines and chemokines associated with the senescence-associated secretory phenotype (SASP). Notably, *Ccl2, Cxcl2*, and *Il6* levels were significantly increased (*p* < 0.05), while *Il1b* showed a substantial increase (*p* = 0.0505) in PTECs cocultured with CD38^hi^ macrophages (Figure 6J). Moreover, when PTECs were incubated with recombinant CD38, we observed a dose-dependent decrease in intracellular NAD^+^ levels (Figure S8A) accompanied by an upregulation of SA-β-gal activity (Figure S8B). Consistently, recombinant CD38 treatment led to increased levels of phosphorylated nuclear histone H2A.X (p-H2A.X), p16^INK4A^, and p21^CDKN1A^, while reducing the levels of the anti-aging proteins sirtuin1 and sirtuin3 in PTECs (Figures S8C and S8D). These findings collectively suggest that CD38^hi^ macrophages directly induce senescence in renal tubular cells.

### Macrophage-specific knockout of *Cd38* attenuates RTEC senescence and renal chronic inflammation

**Figure 7:**
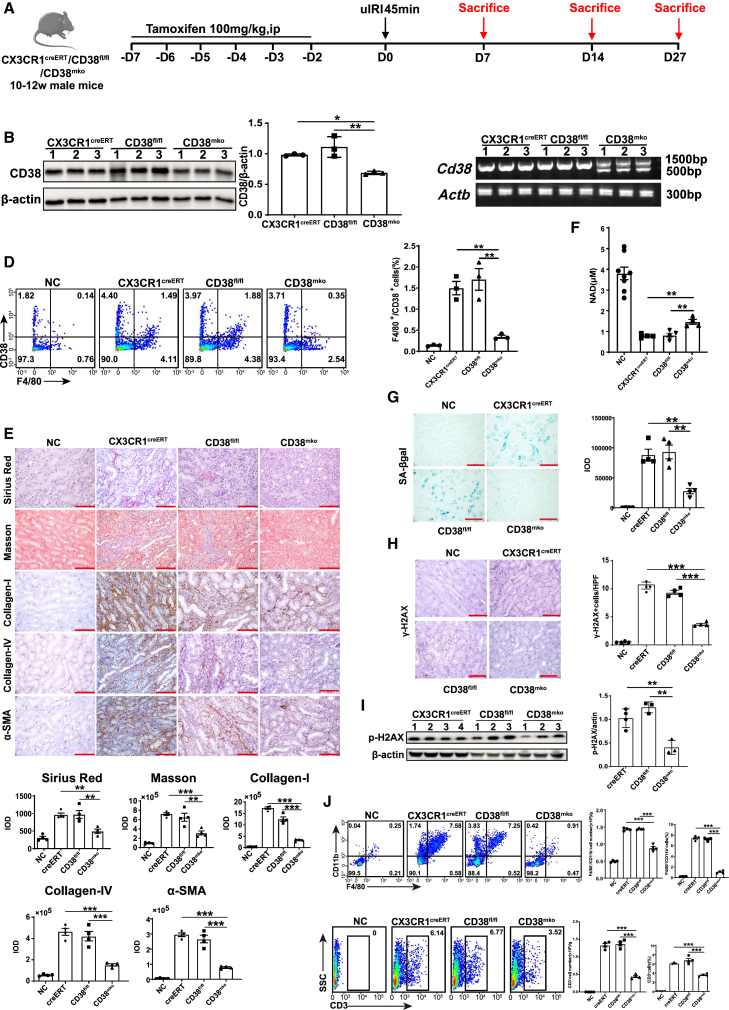
Macrophage-specific knockout of *Cd38* attenuates RTEC senescence and renal chronic inflammation (A) Flow chart of tamoxifen induction protocol in uIRI animal model. CD38^mko^, mice with macrophage specific knockout of CD38; CD38^fl/fl^, CX3CR1^creERT^, control mice for CD38^mko^. (B–D) Validation of *Cd38* knockout. (B) Western blotting of CD38 from kidneys on day 7 post-uIRI and the quantification results. *n* = 3; ∗*p* < 0.05 and ∗∗*p* < 0.01. (C) The kidney CD38 gene floxed fragment assayed by agarose gel electrophoresis. Floxed short DNA fragment (679 bp) was amplified by PCR in CD38^mko^ group. (D) Flow cytometry plot showing the number of CD38^hi^ macrophages at day 14 post-uIRI. *n* = 3; ∗∗*p* < 0.01. (E) Representative images of Sirius Red, Masson, collagen-I, collagen-IV, and α-SMA staining of kidney sections at day 14 post-uIRI and semi-quantitative analysis of IOD in each indicated group. Ten images were taken for each sample. *n* = 3 for each indicated group; ∗∗*p* < 0.01 and ∗∗∗*p* < 0.001. (F) Quantification of NAD^+^ level in kidney samples collected from NC or at day 14 post-uIRI. Sham operated right kidney as normal control (NC). *n* = 8 for NC, and *n* = 4 for CX3CR1^creERT^, CD38 ^fl/fl^, and CD38^mko^ group; ∗∗*p* < 0.01. (G) Representative images of SA-β-gal staining of kidney sections at day 14 post-uIRI and semi-quantitative analysis in each indicated group. *n* = 4; ∗∗*p* < 0.01. (H) Representative images of γ-H2AX staining of kidney sections at day 14 post-uIRI and semi-quantitative analysis in each indicated group. *n* = 4 for each indicated group; ∗∗∗*p* < 0.001. (I) Western blotting of kidney p-H_2_AX and the quantification of expression level at day 14 post-uIRI. *n* = 4 for CX3CR1^creERT^ group, and *n* = 3 for CD38 ^fl/fl^ and CD38^mko^ group; ∗∗*p* < 0.01. (J) Flow cytometry plot showing the percentage of kidney F4/80^hi^/CD11b^hi^ macrophages at day 14 post-uIRI. *n* = 4 for each indicated group; ∗∗∗*p* < 0.001. (K) Flow cytometry plot showing the percentage of CD3^hi^ T cells from kidneys at day 14 post-uIRI. *n* = 4 for each indicated group; ∗∗∗*p* < 0.001. All scale bars, 100 μm. Student’s t test.

To further investigate the role of macrophage CD38 in the progression from AKI to CKD *in vivo*, we generated a mouse model with inducible macrophage-specific ablation of *Cd38* by crossbreeding *Cx3cr1-CreERT2* mice with *Cd38 fl/fl* mice. *Cx3cr1-CreERT2* mice, *Cd38 fl/fl* mice, and *Cd38 fl/fl*, *Cx3cr1-CreERT2* mice received six consecutive intraperitoneal injections of tamoxifen before being subjected to uIRI (Figure 7A). Western blotting analysis of kidney lysates from *Cx3cr1-CreERT2* (CreERT), *Cd38 fl/fl* (CD38^f/f^), and *Cd38 fl/fl*, *Cx3cr1-CreERT2* (CD38^mko^) mice on day 7 post-uIRI revealed a 45% reduction in CD38 protein levels in the CD38^mko^ group (Figure 7B). Analysis of DNA isolated from the kidneys of CreERT, CD38^mcon^, and CD38^mko^ mice at day 14 following uIRI demonstrated the presence of a 679-bp recombined fragment, indicating effective genetic ablation of *Cd38* expression in the CD38^mko^ mice (Figure 7C).

The number of CD38^hi^ macrophages in the injured kidneys of CD38^mko^ mice was significantly decreased at day 14 after uIRI compared to that in the CreERT or CD38^f/f^ mice (Figure 7D). Histological examination on kidney tissues at day 14 after uIRI revealed interstitial extracellular matrix deposition in the kidneys of CreERT or CD38^f/f^ mice following uIRI; however, these changes were ameliorated in the CD38^mko^ kidneys (Figure 7E). Renal NAD^+^ levels were decreased in the CreERT or CD38^f/f^ kidneys after uIRI, but were partially restored in the CD38^mko^ kidneys (Figure 7F). In addition, immunohistochemical staining and western blotting showed reduced levels of SA-β-gal and phosphorylated γ-H2AX in the CD38^mko^ kidneys compared to those in the fibrotic CreERT or CD38^f/f^ kidneys (Figures 7G–7I). Moreover, flow cytometric analysis revealed a significant reduction in immune cell infiltration in the CD38^mko^ kidneys, specifically F4/80^+^ macrophages and CD3^+^ T lymphocytes (Figures 7J and 7K). Collectively, these results suggested that the deletion of *Cd38* in macrophages inhibits tubular cell senescence and attenuates kidney fibrosis following IRI.

Kidney tissues from CreERT, CD38^f/f^, and CD38^mko^ mice were also collected on day 27 post-uIRI. The percentage of CD38^hi^ macrophages remained lower in the injured kidneys of CD38^mko^ mice compared to those of CreERT or CD38^f/f^ mice (Figure S9A). Renal NAD^+^ levels were partially restored following CD38 knockdown (Figure S9B). Consequently, the CD38^mko^ kidneys exhibited reduced SA-β-gal staining and less interstitial fibrosis (Figures S9C and S9D).

To further assess the impact of CD38^hi^ macrophages on renal function, bilateral ischemia reperfusion (bIRI) was induced in both C57BL/6j mice and mice with *Cd38* gene modification. In bIRI mouse model using C57BL/6j mice, serum creatinine levels increased on day 1 post-injury and did not return to baseline until 45 days after IRI, thereby confirming the establishment of a non-repairable IRI model (Figure S10A). CD38^hi^ macrophages also began to emerge on the third day after surgery and persisted into the chronic phase, consistent with findings from the uIRI model (Figures S10B and S10C).

The effects of *Cd38* knockout in macrophages on renal function and renal fibrosis were also evaluated in the bIRI model (Figure S11A). Postoperative serum creatinine and urea nitrogen levels at different time points are shown in Figure S11B. Notably, while the knockdown of *Cd38* in macrophages did not influence the decline in renal function during the acute phase, it facilitated recovery of renal function during the later stages (days 5, 7, and 10) following reperfusion injury. Furthermore, renal fibrosis was attenuated in the CD38^mko^ kidneys, paralleling observations made in the uIRI mouse model (Figure S11C).

### Inhibition of CD38 by 78c alleviates fibrosis progression in uIRI or UUO models

**Figure 8:**
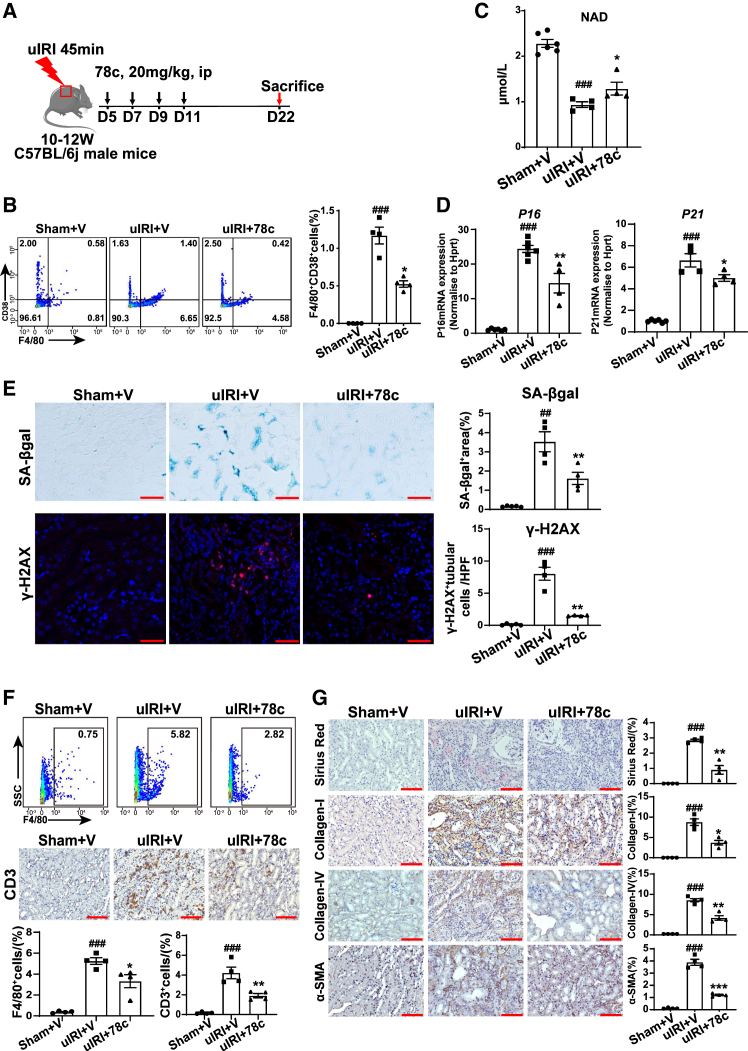
Inhibition of CD38 by small molecular inhibitor 78c alleviates fibrosis progression in the uIRI model (A) Flow chart of drug treatment strategy in uIRI mouse model. (B) Flow cytometry plot showing the percentage of kidney CD38^hi^ macrophages on day 22 post-uIRI. *n* = 4 for each indicated group. ###*p* < 0.001 compared to sham+vehicle group, and ∗*p* < 0.05 compared to uIRI+V group. (C) Quantification of kidney NAD^+^ level on day 22 post-uIRI. *n* = 6 for sham, and *n* = 4 for uIRI+V and uIRI+78c. ###*p* < 0.01 compared to sham+V group, and ∗*p* < 0.05 compared to uIRI+V group. (D) Relative *p16*, *p21* mRNA levels in the kidneys on day 22 post-uIRI. *n* = 4 for sham+V, *n* = 6 for uIRI+V, and *n* = 4 for uIRI+78c. ###*p* < 0.001 compared to sham+V group, and ∗*p* < 0.05 and ∗∗*p* < 0.01 compared to uIRI+V group. (E) Representative images of SA-β-gal and γ-H2AX staining of kidney sections on day 22 post-uIRI. *n* = 5 for sham+V, and *n* = 4 for uIRI+V and uIRI+78c. ###*p* < 0.001 compared to sham+vehicle group, and ∗∗*p* < 0.01 compared to uIRI+V group. (F) Flow cytometry plot of F4/80^+^ macrophage and representative images of CD3 immunohistochemical staining from kidneys on day 22 post-uIRI. *n* = 4 for each indicated group. ###*p* < 0.001 compared to sham+vehicle group, and ∗∗*p* < 0.01 compared to uIRI+V group. (G) Representative images of Sirius red, collagen-I, collagen-IV, and α-SMA staining of kidney sections on day 22 post-uIRI. *n* = 4 for each indicated group. ###*p* < 0.001 compared to the sham+V group, ∗*p* < 0.05, ∗∗*p* < 0.01, and ∗∗∗*p* < 0.001 compared to uIRI+V group. All scale bars, 50 μm. V, vehicle.

To investigate the therapeutic effect of CD38 inhibition, we initiated intraperitoneal administration of small molecular inhibitor 78c at a dose of 20 mg/kg/day, starting on day 5 post-uIRI and continuing with 4 doses administrated on alternate days (days 5, 7, 9, and 11). Mice were euthanized on day 22 post-uIRI, and kidney tissue samples were collected (Figure 8A). We observed an increase in the number of CD38^hi^ macrophages in the kidneys at day 22 after uIRI, which was attenuated by the administration of 78c (Figure 8B). Treatment with 78c significantly mitigated the reduction in NAD^+^ content in the uIRI kidney (Figure 8C). Furthermore, the post-injury cellular senescence phenotype was assessed through quantitative real-time reverse transcription PCR analysis of *p16* and *p21* (Figure 8D); as well as via anti-γ-H2AX and SA-β-gal staining (Figure 8E). The infiltrations of F4/80^+^ macrophages and CD3^+^ T cells (Figure 8F), along with the extent of kidney fibrosis, analyzed through Sirius red staining and immunohistochemical staining for anti-collagen I, collagen IV, and anti-α-smooth muscle actin (α-SMA) (Figure 8G), were all significantly reduced following 78c treatment. Subsequently, we examined the effects of CD38 inhibition on renal fibrosis using the same dosing regimen for 78c, but varying the initiation time to immediately post-uIRI (D0), 3 days post-uIRI (D3), 7 days post-uIRI (D7), or 10 days post-uIRI (D10). Initiation of 78c treatment on D0, D3, D5, or D7 significantly alleviated the extent of renal fibrosis in the uIRI mice, whereas initiation on D10 did not yield a significant effect (Figure S12).

The effect of CD38 inhibition was further explored in a UUO mouse model. In this context, 78c (20 mg/kg/day) was administered on days 3, 4, 6, and 8 following the UUO procedure (Figure S13A). Mice were euthanized on day 14 post-surgery, and kidney tissue samples were collected. Administration of 78c tended to decrease the number of infiltrated F4/80^+^ macrophages (Figure S13B) and CD3^+^ T cells in the kidney (Figure S13C). Sirius red staining and anti-collagen I staining revealed a significantly reduced degree of renal fibrosis in the UUO mice treated with 78c (Figure S13D). Collectively, these findings indicate that the application of the CD38-specific inhibitor 78c effectively inhibits the progression of renal fibrosis in both ischemic and obstructive injury models.

## Discussion

Chronic renal fibrosis resulting from a maladaptive response to AKI is a fundamental contributor to the progression of CKD and the development of ESRD.^23^ Macrophages exhibiting diverse phenotypes are known to play multifaceted roles in renal injury, inflammation, repair, and fibrosis, and they serve as crucial mediators in the transition from AKI to CKD.^24^ However, the heterogeneity of macrophages during this transition remains inadequately understood, which complicates efforts to implement targeted interventions against specific profibrotic macrophage subsets for the treatment of AKI.

Taken advantages of scRNA-seq technology, we investigated macrophage heterogeneity throughout the progression from AKI to CKD by analyzing data derived from a model of uIRI in mice. Our analysis revealed a distinct subset of macrophages, originating from KRMs, characterized by high expression of the CD38 marker gene. Csf1 is known to promote the proliferation and differentiation of tissue-resident macrophages, including those in pulmonary and renal contexts.^25^^,^^26^ Actually, we observed that CD38^hi^ macrophages in injured kidneys were in a proliferative state. Inhibition of Csf1 signaling using the Csf1r inhibitor pexidartinib significantly reduced the proportion of CD38^hi^ macrophages, while blockade of monocyte infiltration via CCR2 neutralizing antibodies did not affect the emergence or maintenance of CD38^hi^ macrophages. These findings underscore the pivotal role of Csf1/Csf1r pathways in the differentiation of KRMs into CD38^hi^ macrophages. The biological functions of these CD38^hi^ macrophages were primarily associated with tissue aging and chronic inflammation. Notably, the abundance of CD38^hi^ macrophages exhibited a significant correlation with the extent of renal fibrosis, both in injured murine kidneys and in AKI patients.

In previous studies, we explored the role of IMs in the initiation and amplification of inflammation during the acute phase of IRI-AKI.^11^ The physiological and pathological roles of resident macrophages in maintaining tissue homeostasis and promoting maladaptive response to injury have garnered considerable interest.^27^^,^^28^ It has been proposed that macrophages contribute to renal fibrosis either through direct activation of interstitial fibroblasts via cytokine secretion (e.g., IL-1, MMP2, MMP12, galectin-3, and PDGF) or through a process known as macrophage-to-myofibroblast transition (MMT).^29^^,^^30^^,^^31^^,^^32^^,^^33^^,^^34^ For instance, a population of profibrotic macrophages characterized by the expression of *Spp1*, *Fn1*, and *Arg1* (termed Spp1 macrophages) has been shown to expand in both human CKD and heart failure, differentiating in response to CXCL4 signaling from platelets and orchestrating fibroblast activation through a network of Spp1, Fn1, and Sema3 interactions.^35^ Similarly, in another UUO animal model, a population of Arg1^+^ macrophages have been suggested to originate from infiltrated monocytes and contribute to renal fibrosis through signaling via Fn1-integrin, Pdgfa-Pdgfrß, and Tnf-Tnfsfr1 pathways toward mesenchymal cells.^36^ However, our current study indicates that the contribution of CD38^hi^ macrophages to renal fibrosis may be mediated by the protein-coding gene CD38 itself.

CD38 is not only a cell surface glycoprotein but it also serves as a multifunctional extracellular enzyme, with its primary enzymatic activity being the hydrolysis of NAD^+^.^37^ NAD^+^ and its reduced form, NADH, are critical for energy metabolism and fatty acid oxidation in RTECs through the regulation of redox reactions, facilitating ATP production.^38^ Additionally, NAD^+^ acts as a substrate for enzymes involved in DNA damage repair and aging, such as sirtuins and poly (ADP-ribose) polymerases.^39^ Recent studies have reported a decline in kidney NAD^+^ levels in models of cisplatin-induced and IRI-induced AKI, with restoration of NAD^+^ levels providing protective effects against kidney damage.^40^^,^^41^^,^^42^^,^^43^ Consequently, we hypothesized that the CD38^hi^ macrophages identified in our MPC atlas may promote a senescent phenotype in RTECs by depleting NAD^+^ within renal tissues, thereby driving the development of fibrosis and chronic inflammation. To test our hypothesis, we evaluated CD38 protein levels in the kidneys following IRI. Notably, CD38 protein levels reached their peak during the chronic phase following AKI. The abundance of CD38^hi^ macrophages and CD38 protein levels exhibited a negative correlation with renal tissue NAD^+^ levels, while they positively correlated with the expression of cellular senescence markers. Importantly, sorted CD38^hi^ macrophages from injured kidney tissues demonstrated the capacity to diminish extracellular NAD^+^ levels and induce a senescent phenotype in primary cultured TECs, as evidenced by increased expression of SA-β-gal and elevated levels of SASP cytokines/chemokines, including Ccl2, Cxcl2, and Il6. Furthermore, the application of recombinant CD38 to PTECs resulted in increased expressions of pH2AX, p16, and p21, alongside the inhibition of anti-aging proteins sirtuin1 and sirtuin3. *In vivo* investigations revealed that specific knockdown of *Cd38* in macrophages could restore NAD^+^ levels in injured kidney tissues, inhibit RTEC senescence, and alleviate fibrosis. Additionally, the infiltration of inflammatory immune cells, specifically F4/80^+^ macrophages and CD3^+^ T cells, was significantly reduced following *Cd38* knockdown. These results collectively support the hypothesis that CD38^hi^ macrophages play a crucial role in driving the progression of renal fibrosis and chronic inflammation by secreting the NAD^+^-depleting enzyme CD38, which fosters a senescent phenotype in RTECs. The complexity of cellular interactions within the CD38^hi^ macrophage-organized microenvironment in the kidney, particularly how senescent RTECs release inflammatory factors that attract additional immune cells, and how the intricate dynamics between CD38^hi^ macrophages and other immune cell populations, necessitates further investigation.

Current therapeutic approaches aimed at enhancing NAD^+^ levels to prevent cellular senescence and tissue aging focus on stimulating NAD^+^ production or mitigating its loss by inhibiting enzymatic degradation or consumption. In this study, through association analysis, *in vitro* CD38^hi^ macrophage and PTEC coculture, and *in vivo* CD38-specific knockout in macrophages, we demonstrated that CD38 may represent a compelling therapeutic target for the treatment of AKI. Recently, thiazoloquin(az)olin(on)es, such as the small molecule 78c, have been identified as potent inhibitors of CD38 (CD38i)^44^^,^^45^^,^^46^ with high specificity and potency. In our research, we established the first dosing regimen for 78c in mouse models of renal fibrosis (uIRI and UUO) and validated its efficacy in alleviating the chronic progression of AKI and renal fibrosis. Our data demonstrate that increasing tissue NAD^+^ levels through the administration of 78c during the early stage of injury, before the establishment of fibrosis, mitigates several senescent characteristics of renal tubules, reduces inflammatory cell infiltration, and alleviates renal fibrosis. Given the reversible nature of 78c’s inhibition, and that the route of administration for 78c was intraperitoneal, further investigation is needed into the combination of 78c with NAD^+^ precursors such as β-Nicotinamide Mononucleotide (NMN) and Nicotinamide Riboside (NR), as well as the possibility of using an oral dosage of 78c for the treatment of renal fibrosis. Notably, CD38 inhibition in humans has been pursued using CD38-specific blocking antibodies.^47^^,^^48^ Antibodies such as daratumumab and isatuximab have received approval from the US Food and Drug Administration for the treatment of certain human malignancies.^49^^,^^50^ The potential of CD38-specific antibody blocking/inhibition warrants further investigation for its translational application in addressing the chronic progression of AKI. In recent years, there has been significant interest in targeted drug delivery and gene intervention strategies specifically aimed at macrophages.^51^^,^^52^ The development of macrophage-targeted *CD38* gene knockout nanomedicine, as well as the use of CD206-conjugated nanoparticles for the targeted delivery of 78c to macrophages, warrants further investigation.

In summary, we have identified a previously unrecognized subset of CD38^hi^ macrophages with pathogenic roles in the progression from AKI to CKD via dysregulation of NAD^+^ homeostasis. Genetic targeting of macrophage CD38 or pharmacological inhibition of its activity resulted in increased NAD^+^ levels, leading to the attenuation of renal tubular senescence, fibrotic responses, and chronic inflammation in preclinical mouse disease models. Therefore, interventions aimed at modulating CD38-mediated NAD^+^ metabolism may represent a novel therapeutic strategy for ameliorating chronic renal fibrosis induced by acute injury.

## Materials and methods

The antibodies and materials used herein are listed in Table S4.

### Animals

Specific pathogen-free (SPF) grade C57BL/6J male mice (10–12 weeks, 25–30 g weight) were purchased from SPF and HFK Biotechnology Co., Ltd. (Beijing, China) and bred in a pathogen-free environment at the Peking University First Hospital Animal Center. *Cx3Cr1-CreERT2* and *Cd38 fl/fl* mice were generated by Shanghai Nanfang Research Center for Model Organisms (Shanghai, China). CD38^mko^ (*CD38 fl/fl, Cx3cr1-CreERT2*) mice were generated by mating *Cx3Cr1-CreERT2* mice with *Cd38 fl/fl* mice. The detailed process is shown in Figure S14. The plans and procedures for the animal experiments were approved by the Experimental Animal Welfare Ethics Committee of Peking University First Hospital (approval nos. J202134 and J2022128).

### Patients with AKI

Patients who were hospitalized in the Renal Division of Peking University First Hospital from 2006 to July 2020, and underwent renal biopsy with a pathological diagnosis of only acute tubular injury (ATI) were included. Those who had ATI concomitant with glomerular or vascular lesions were excluded. Nine para-carcinoma kidney tissues pathologically identified as the healthy parts of the kidney were used as controls. The protocol concerning the use of patient samples was approved by the Biomedical Research Ethics Committee of Peking University First Hospital (approval no. 2023483). Informed consent was obtained from all participants. The clinical characteristics of the enrolled patients have been previously described^11^ and detailed in Table S5. Briefly, thirty-six patients with AKI were included. The average age of the AKI patients was 44.50 ± 13.60 years (male: 63.9%). The causes of AKI were defined as nephrotoxicity in 25 patients and ischemic injury in 8 patients, while unknown etiology in the other 3 patients. The serum creatinine levels were of 664.00 ± 446.00 μmol/L at peak and 315.60 ± 273.10 μmol/L at biopsy. The severity of tissue injury was assessed with semi-quantitative pathological score (0–4), and 16 cases (44.4%) were defined as mild pathological injury, while 20 cases (55.6%) as severe injury.

### uIRI/bIRI/UUO mouse models

Renal uIRI/bIRI/UUO were performed as described previously.^11^^,^^53^ Briefly, mice were anesthetized by intraperitoneal (i.p.) injection of 0.5% sodium pentobarbital. For uIRI, the left kidney was exposed and the renal pedicle was clamped using a vascular clip (Roboz Surgical Instrument Co, Gaithersburg, MD, USA) for 45 min. For bIRI, both kidneys were exposed and the renal pedicles were clamped for 25 min. The mice were kept at a constant body temperature of 37°C. For UUO, the back skin of the mouse was cut open. The left ureter was isolated and double-ligated using 6-0 silk, and the ureter was cut between the two ligation points. In the sham group, only anesthesia and muscle incision were performed.

### Assessment of macrophage-specific *Cd38* gene ablation in the kidney tissues

*Cd38 fl/fl* mice were generated by Shanghai Nanfang Research Center for Model Organisms. *Cd38* flox allele was detected by primers: F: 5′ATGGGGACACAGAGGAAAGAC3′ and R: 5′ACTCAAAGGACCGAACACTG3′. CD38^mko^ (*CD38 fl/fl, Cx3cr1-CreERT2*) mice were generated by mating *Cx3Cr1-CreERT2* mice with *Cd38 fl/fl* mice. Cre recombinase activity was induced by i.p. injection of tamoxifen according to strategies shown in Figures 7A and S11A. To detect the active Cre recombination in the kidney, genomic DNA was extracted from kidney tissues of *Cx3cr1CreERT2*, *Cd38* ^fl/fl^, and *CD38*^*mko*^ mice using a DNA animal tissue extraction kit (Tiangen Biotech Co. Ltd., Beijing, China). DNA (1 μg) was used for PCR amplification by the KOD -Multi & Epi- system (Toyobo, Osaka, Japan; cat. no: KME-101). Primer pair F: 5′CCCACCTGCAGTAACCCTAC3′ and R: 5′GGGTTTGGGGGCTTCACTTA3′ was used to amplify the recombined region. The resulting PCR products were subjected to agarose gel electrophoresis. The PCR product from *Cd38* allele with Cre recombination was 679 bp, while that without Cre recombination was 1,558 bp.

### Preparation of kidney mononuclear phagocytes for scRNA sequencing

Whole kidney single-cell suspensions were prepared as previously described.^11^ In brief, the kidney was excised and placed in cold 1640 medium (Gibco, Grand Island, NY, USA). The organ was then cut into 1 mm^3^ fragments on ice and incubated in 5 mL of digestion buffer containing 0.25 mg/mL Liberase TH (Thermolysin High) (Roche, Indianapolis, IN, USA) and 50 μg/mL DNase I (NEB, Ipswich, MA, USA) at 37°C for 30 min. Following digestion, the resultant cell suspension was filtered through a 40 μm cell strainer, centrifuged at 400 × g at 4°C for 5 min, and resuspended in 500 μL of pre-chilled phosphate-buffered saline (PBS) containing 0.04% BSA. Dead cells were removed using the Dead Cell Removal Kit (Miltenyi Biotec, Germany, 130-090-101), and viable cells were collected.

Single-cell suspensions were stained in fluorescence-activate cell sorting (FACS) buffer (PBS, 1% BSA, 0.05% sodium azide) with Calcein AM, 7-AAD, and fluorophore-conjugated antibodies against CD45, CD11b, and F4/80. Forward scatter (FSC) and side scatter (SSC) parameters were used to eliminate cell debris, while SSC or FSC weight (W) and height (H) parameters were used to exclude cell aggregates. 7-AAD and Calcein AM were used to identify and exclude dead cells. CD45^+^ cells were initially gated, following by sorting of either CD11b^+^ or F4/80^+^ cells using an Aria SORP cell sorter (BD Biosciences, San Jose, CA, USA). The specific gating strategies were illustrated in Figure S1D. Six mice were used for each time point. To obtain sufficient number of MPC for analysis, while also including monocyte-derived macrophages (CD11b^hi^F4/80^low^) to trace the developmental trajectory of kidney macrophages after injury, the sorted F4/80^+^ cells and CD11b^+^ cells were combined at a 1:1 ratio for subsequent scRNA-seq.

### scRNA-seq by 10× Genomics and data processing

scRNA-seq and data processing were performed as previously described.^11^ Briefly, FACS-sorted cells were resuspended to a final cell concentration of 700–1,200 cells/μL with more than 85% viability, as determined by Countess II (Thermo Fisher Scientific, Waltham, MA, USA). Cells (8,000–1,2000) were captured in droplets (10**×** Genomics Chromium Single Cell 3ʹ Reagent Kits; 10**×** Genomics, Pleasanton, CA, USA). After the reverse transcription, 50 ng of amplified cDNA was fragmented, end-repaired, and sequenced on an Illumina platform (Illumina, San Diego, CA, USA) using 150 paired-end reads at a coverage of 40,000 mean reads per cell. Raw sequencing data were analyzed following the standard Chromium’s Cell Ranger pipeline (10**×** Genomics) to align the raw sequence reads according to the reference genome (mm10–2.1.0) using STAR. The count matrices from different batches were merged, integrated, and processed with the Seurat pipeline.^54^ For quality control, cells with a mitochondrial gene percentage less than 5%, unique gene counts between 1,000 to 50,000, and detected genes between 200 and 7,000 were retained. The merged filtered count matrix was then normalized and scaled using the default SCTransform pipeline.^55^ Principal component analysis (PCA) was performed based on the 3,000 highly variable genes detected in the Single-Cell Transcriptomics (SCT) assay. The standard harmony integration pipeline was used to remove the batch effect^56^; 38,074 sorted cells passed quality control, and 27,544 cells were selected from these sorted cells as mononuclear phagocytes. According to PCA, the first 30 integrated principal components were selected as input for UMAP reduction and unsupervised clustering. Based on the Clusteree package analysis results and biological significance, a resolution of 0.5 was chosen as the MPC clustering resolution. The Seurat R package was used to identify differentially expressed genes (DEGs) and to visualize gene expression.^54^

### Gene set scoring and comparison in the scRNA-seq data

Gene sets containing related markers were constructed based on previously published reports on different lineages, and cellular functions of MPCs (Table S3). Gene set scores of each single cell were calculated using the “AddModuleScore()” function in the Seurat package, and the gene set scores were compared within each class following the frame same as the “CellCycleScoring()” function. This algorithm assigned the predominant phenotype to single cells according to the score difference between gene sets.

### RNA velocity analysis

RNA velocity analysis to distinguish spliced and unspliced RNA molecules was performed using scVelo and followed the standard pipeline of the scVelo python package to calculate and visualize the RNA velocity of the scRNA-seq data.^57^^,^^58^^,^^59^^,^^60^ For further details, please refer to our previous article for the specific methods.^11^

### Gene clustering based on pseudo-temporal expression patterns

Gene clustering based on pseudo-temporal expression pattern analysis used a previously published method.^11^ Briefly, genes whose expression changed significantly during the development and transition process were identified using the scVelo package. The Monocle package was used to cluster and plot dynamic genes along the pseudotime line.^61^ Subsequently, the obtained gene modules were analyzed and compared using GOBP comparison analysis.

### Flow cytometry analysis

A single-cell suspension of the entire kidney was prepared as previously described.^11^ The single-cell suspensions were incubated directly with 7-AAD and fluorescence-labelled antibodies, specifically anti-F4/80, anti-CD38, anti-CD11b, and anti-CD3, as detailed in Table S4. The samples were acquired using FACS Verse and Aria II instrument (BD Biosciences) and subsequently analyzed with Flow Jo version 10 (Treestar, Ashland, OR, USA).

For BrdU labeling, mice were administered 100 mg/kg BrdU 24 h prior to sacrifice. Following the dissociation of the entire kidney, the single cells were fixed using Fixation Buffer (BioLegend) and permeabilized with Wash Buffer (BioLegend). Subsequently, the kidney cells were stained with the following antibodies: Alexa Fluor 488-conjugated anti-Ki67 (D3B5), Alexa Fluor 488-conjugated anti-BrdU (BU1/75(ICR1)), PE-Cy7-conjugated anti-F4/80 (BioLegend), and APC-conjugated anti-CD38 (BioLegend). After a 30-min incubation at 4°C, the samples were washed and analyzed on the BD FACSVerse (BD Biosciences, USA).

The Fluorescence Minus One (FMO) controls for flow cytometry experiments were shown in Figure S15.

### PTEC isolation and culture

The primary PTEC isolation and culture were carried out as previously described.^62^ In brief, kidneys from wild-type C57BL/6j mice were extracted and washed with physiological saline. The kidneys were cut into approximately 1 mm^3^ pieces and digested in 2 mg/mL collagenase I and collagenase II at 37°C for 25 min. Enzyme digestion was stopped by adding equal volume of Dulbecco’s modified Eagle’s medium (DMEM)/F12 medium. The kidney cell suspension was filtered through a 70-μm cell filter (Millipore, Billerica, MA, USA) and then centrifuged at 300 × *g* for 5 min at 4°C. The cell pellet was resuspended in pre-prepared K1 medium: DMEM/F12 supplemented with 25 ng/mL epidermal growth factor, 1 ng/mL prostaglandin E1, 5 × 10^−11^ mol/L triiodothyronine, 5 × 10^−8^ mol/L hydrocortisone (Sigma-Aldrich, St. Louis, MO, USA), insulin-transferrin-selenium culture supplement, 1% penicillin/streptomycin, 25 mM HEPES, and 5% fetal bovine serum (FBS) I (Invitrogen, Waltham, MA, USA). The cells were then placed on culture dishes and incubated at 37°C in a humidified incubator containing 5% CO_2_ and 95% air for further cultivation for 5–7 days before the experiments.

### PTEC and macrophage coculture

CD38^hi^macrophages (CD38^hi^CD45^+^F4/80^+^) and CD38^low^ macrophages (CD38^low^CD45^+^F4/80^+^) were isolated from kidney tissues following uIRI using flow cytometry. A single-cell suspension of the entire kidney was prepared as described above. The cell pellets were subsequently incubated with fluorescence-labeled antibodies, including PE anti-mouse CD45, PE-Cy7 anti-mouse F4/80, and APC anti-mouse CD38, and sorted utilizing the Aria II instrument (BD Biosciences); 1×10^4^ CD38^hi^ macrophages and CD38^low^ macrophages were collected in 0.5 mL of DMEM/F12 medium supplemented with 1% FBS. Following centrifugation to remove the collection medium, the macrophages were seeded on 1×10^6^ adherent PTECs in a single well of a 6-well plate. The coculture was maintained in K1 medium for a duration of two days.

### Immunohistochemistry and immunofluorescence

Immunohistochemistry and immunofluorescence assays were performed as described previously.^11^ Briefly, the paraffin-fixed sections were subjected to gradient ethanol dewaxing and antigen repair (Tris/EDTA, PH = 9.0). For immunohistochemistry, the sections were blocked with peroxidase-blocking buffer (Zhong Shan Jin Qiao, Beijing, China) for 20 min at room temperature (RT) and 3% BSA for 30 min at 37°C, and then incubated with the primary antibodies (α-SMA, anti-collagen I, anti-collagen IV, anti-CD3, anti-F4/80, anti-γ-H2AX, anti-P16, and anti-P21) (Table S1) at 4°C overnight. Subsequently, secondary antibodies (Zhong Shan Jin Qiao) were applied and detection was performed using 3,3′-diaminobenzidine (DAB). Anti-γ-H2AX, anti-F4/80, anti-CD38, anti-CSF1, and anti-CD68 antibodies (Table S4) were used for immunofluorescence. Primary antibodies were incubated with tissues overnight at 4°C, and followed by fluorophore-conjugated secondary antibody incubation. Ten random visual fields in both the cortex and medulla region were acquired on a DM2500 light macroscope (Leica, Wetzlar, Germany) or a Zeiss LSM 780 confocal microscope (Carl Zeiss, Berlin, Germany). All images were acquired using the same microscope and camera set. The number of CD38^+^ macrophages was quantified in 10× high-powered fields (HPF, 400× magnification) in de-identified samples by two researchers. The intensities of the positive staining in the cytoplasm and membranes were determined using the integrated optical density (IOD) per area of tissue (400× magnification) and measured using Image Pro Plus software (Media Cybernetics, Rockville, MD, USA).

### Mouse serum creatinine and urea quantification

Fresh mouse blood samples were centrifuged at 2500 rpm at 4° for 10 min. Serum creatinine levels were detected using the Quantichrom Creatinine Assay Kit (DICT-500; BioAssay Systems) as the manufactural instructions. Mouse serum creatinine and urea (BUN) was measured using a DIUR-500 assay kit (Bioassay Systems, Hayward, CA) as the manufactural instructions.

### Quantification of total NAD concentration in kidney tissues

NAD levels in kidney tissues were measured using the NAD^+^/NADH Quantification Kit (Beyotime, Jiangsu, China; S0175). Briefly, 20 mg of kidney tissue was homogenized in 400 μL of NAD^+^/NADH extraction solution provided in the kit. The homogenate was then centrifuged at 12,000 × g for 5–10 min at 4°C, and 20 μL of the supernatant was collected for analysis. The samples were incubated with 90 μL of ethanol dehydrogenase working solution for 10 min at 37°C in the dark. Absorbance was measured at 450 nm using a Promega GloMax luminometer (Mannheim, Germany). An NAD concentration standard curve was generated according to the manufacturer’s instructions, and the total NAD concentration in the samples was calculated based on this curve.

### Kidney CD38 quantification

The concentration of CD38 in mouse kidney tissue was quantified using an ELISA with the Mouse CD38 Quantikine Kit (Shanghai Kepeirui Biological Technology Co., Ltd., Shanghai, China), following the manufacturer’s instructions. Briefly, kidney tissue was homogenized in an appropriate volume of saline, and the homogenate was subjected to centrifugation at 3,000 rpm for 10 min. The supernatant was then collected for analysis. A standard curve was generated using the CD38 standard provided in the kit. Standards and samples were incubated with HRP-labeled CD38 antibody for 1 h at 37°C. After the addition of the chromogenic substrate, the optical density (OD) of each well was measured at 450 nm using a Promega GloMax luminometer.

### SA-βgal staining

SA-βgal staining was performed using a Senescence-βgal Staining Kit (Beyotime, C0602) following the manufacturer’s instructions. Briefly, frozen kidney sections and PTECs were fixed with fixative solution for 15 min at RT. Subsequently, the staining working solution (10 μL β-Galactosidase Stain A + 10 μL β-Galactosidase Stain B + 930 μL β-Galactosidase Stain C + 50 μL X-Gal solution) was added and incubated overnight at 37°C. Ten fields of view were randomly selected and photographed under the DM2500 light macroscope. The intensities of positive staining were represented by the IOD per area of tissue (400× magnification), as measured using Image Pro Plus software.

### Kidney histopathological staining

PBS-perfused kidneys were fixed in 10% formalin, sectioned, and stained with (PAS, Masson trichrome, and Sirius Red. PAS staining was performed as previously described.^63^ Briefly, after dewaxing and rehydration, 2 μm paraffin sections were stained with PAS solution for 20 min at RT in the dark. Nuclei were counterstained using hematoxylin. The Masson staining assay was performed using a Masson Stain Kit (G1340, Solarbio) according to the supplier’s instructions. Sirius Red staining was performed using a Sirius Red Stain Kit (AG1471, ACMEC, Shanghai, China) according to the supplier’s instructions. Briefly, 2 μm paraffin sections were stained with Sirius Red solution for 2 h. Nuclei were counterstained using hematoxylin. Images were acquired under the Leica DM2500 light microscope.

### Western blotting analysis

After stimulation with recombinant Cd38 protein, RTECs were washed with pre-chilled PBS and lysed in cold radioimmunoprecipitation assay (RIPA) buffer containing protease and phosphatase inhibitors. The animal tissues were lysed in a suitable amount of cold RIPA buffer containing protease and phosphatase inhibitors. Cell lysates and tissue suspensions were incubated at 4°C for 30 min and then centrifuged at 16,000 × g at 4°C for 30 min. The supernatant was collected for protein quantification using a BCA Protein Assay Kit (NCM Biotech, Newport, RI, USA). Equal amounts of cell supernatant were mixed with SDS-PAGE loading buffer, and boiled at 100°C for 5 min. The extracted proteins were separated on a 4%–20% SDS-PAGE gel (ACE, ET15420Gel, Nanjing, China) and then transferred onto a 0.2 μm polyvinylidene fluoride (PVDF) membrane (Thermo Fisher Scientific). The membrane was blocked at RT in 5% non-fat milk for 1 h, and then incubated with primary antibodies as listed in Table S1 overnight at 4°C. After washing, the PVDF membrane was incubated with horseradish peroxidase (HRP)-conjugated secondary antibodies at RT for 1 h, followed by chemiluminescence detection (Millipore). The immunoreactive protein bands were analyzed using ImageJ software (NIH, Bethesda, MD, USA).

### Real-time RT-qPCR

Total RNA from frozen kidney tissues was extracted using a total RNA Extraction Kit (Tiangen Biotech). The RNA concentration and purity were detected using nanodrop-photometric quantification (Thermo Fisher Scientific). cDNA synthesis was performed by using a FastKing RT kit (Tiangen Biotech). The primer sets for the real-time RT-qPCR are listed in Table S6. qPCR was performed using SYBR Green PCR Master Mixture Reagents (Tiangen Biotech) on the Applied Biosystems 7500 real-time PCR detection system (Applied Biosystems, Foster City, CA, USA).

### Statistical analysis

Testing for the normal distribution of numerical data was performed using the Kolmogorov-Smirnov test. Normally distributed data are shown as mean ± the standard error of the mean (SEM), otherwise the data are presented as the median with interquartile range. Comparison of normally distributed numerical parameters was performed using Student’s t test. Comparison of numerical data with skewed distribution was performed using the Kruskal-Wallis test. Comparison of categorical parameters was analyzed using the chi-squared test. The associations between the kidney CD38^+^ macrophages and collagen I, collagen IV, Sirius Red staining, α-SMA, NAD, p16, p21 RNA and IOD, SA-β-gal, F4/80, CD3, and γ-H2AX were analyzed using Pearson correlation analysis. The associations between the kidney CD38 and CD38^+^macrophages, NAD, SA-βgal, and γ-H2AX were analyzed using Pearson correlation analysis. A two-sided *p* value <0.05 was considered as statistically significant. All of the statistical analyses were performed using GraphPad Prism 8.0 (GraphPad Software, Inc., La Jolla, CA, USA).

## Data availability

The data that support the findings of this study are openly available in Gene Expression Omnibus at https://www.ncbi.nlm.nih.gov/geo (GEO accession nos. GSE174324 and GSE239287).

## Acknowledgments

We would like to thank Yinghua Guo at the flow cytometry core (National Center for Protein Sciences of Peking University) for her assistance with flow cytometry experiments. We also thank the native English-speaking scientists of Elixigen Company (Huntington Beach, CA) for editing our manuscript. This work was supported by grants from the 10.13039/501100001809National Natural Science Foundation of China (grant nos. 82130021, 82300759, and 82270709), the 10.13039/501100012166National Key R&D Program of China (grant nos. 2022YFC2502500 and 2022YFC2502502), the National High Level Hospital Clinical Research Funding (High Quality Clinical Research Project of Peking University First Hospital, no. 2022CR83), the Capital’s Funds for Health Improvement and Research (grant no. CFH2022-1-4071), 10.13039/100031233Michigan Medicine-PKUHSC Joint Institute for Translational and Clinical Research (BMU2022JI002), CAMS Innovation Fund for Medical Sciences (grant no. 2019-I2M-5-046), Peking University Medicine Fund for world’s leading discipline or discipline cluster development (BMU2022DJXK004), the Beijing Young Scientist Program (grant no. BJJWZYJH01201910001006), and the 10.13039/501100012226Fundamental Research Funds for the Central Universities (Peking University Medicine Sailing Program for Young Scholars’ Scientific & Technological Innovation, no. BMU2024YFJHMX003).

## Author contributions

W.Y. and M.L. performed the experiments; W.Y. and Z.L. analyzed the scRNA-seq data; C.X., S.S., and L.Q. helped to collect and interpret the data; L.Q. prepared the histological samples; S.W., G.L., and L.J. assessed tissue histology; Y.C. and W.Y. drafted the manuscript; L.Y. conceived and supervised the study; Y.C. and L.Y. reviewed and edited the manuscript. All authors read and approved the final manuscript.

## Declaration of interests

The authors declare no competing interests.

## Supplemental information

Document S1. Figures S1–S15 and Table S5

Table S1. Top 200 DEGs in 13 clusters of all sorted cells

Table S2. Top 200 DEGs in 15 clusters of MPCs

Table S3. Gene sets used for functional characterization and scoring

Table S4. Antibodies and materials

Table S6. RT-qPCR primers

Document S2. Article plus supplemental information
