# Supplementary material for: CD38hi macrophages promote fibrotic transition following acute kidney injury by modulating NAD+ metabolism
Source: Mol Ther. 2025 May 9;33(7):3434–52. doi: 10.1016/j.ymthe.2025.04.039 (PMC12266028; doi:10.1016/j.ymthe.2025.04.039)
Supplement: Document S1. Figures S1–S15 and Table S5 [file mmc1.pdf]

YMTHE, Volume *33*

## Supplemental Information

# CD38$^{hi}$ macrophages promote fibrotic transition following acute kidney injury by modulating NAD$^+$ metabolism

**Weijian Yao, Menghan Liu, Zehua Li, Lei Qu, Shuang Sui, Chengang Xiang, Lei Jiang, Suxia Wang, Gang Liu, Ying Chen, and Li Yang**

# Supplemental Figures

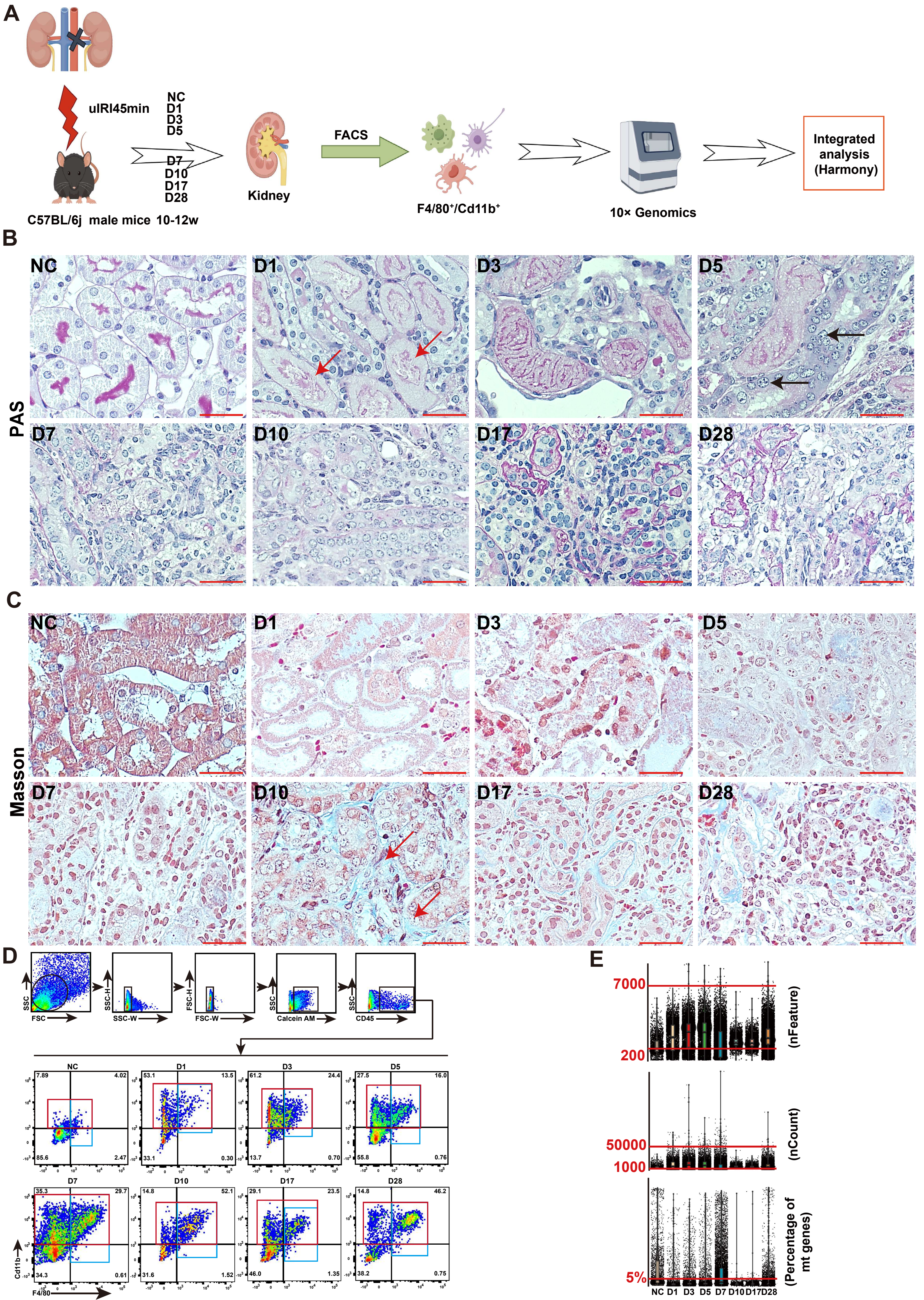

**Figure S1. Sorting strategies of kidney cells from uIRI animals and quality control for sequenced data.** (A) Flow chart of the single cell sequencing experimental design; n = 6 mice at each time point. The flow chart was drawn using Figdraw. (B,C) Representative images of PAS staining (B) and Masson staining (C) on renal tissues from NC and tissues at each time point post-uIRI. Scale bar, 50 μm. Red arrows indicate tubular epithelial cell death in (B). Black arrows indicate tubular epithelial cell division in (B). Red arrows indicate fibrosis in the renal interstitium in (C). Scale bar, 50 μm. (D) Representative flow cytometry gating strategies of kidney cells collected from NC kidneys and kidneys at each time point post-uIRI. (E) The cut-off values of feature, count and percentage of mitochondria genes for seRNA-seq quality control. NC, normal control; PAS, periodic acid-schiff; FSC, forward scatter; SSC, side scatter.

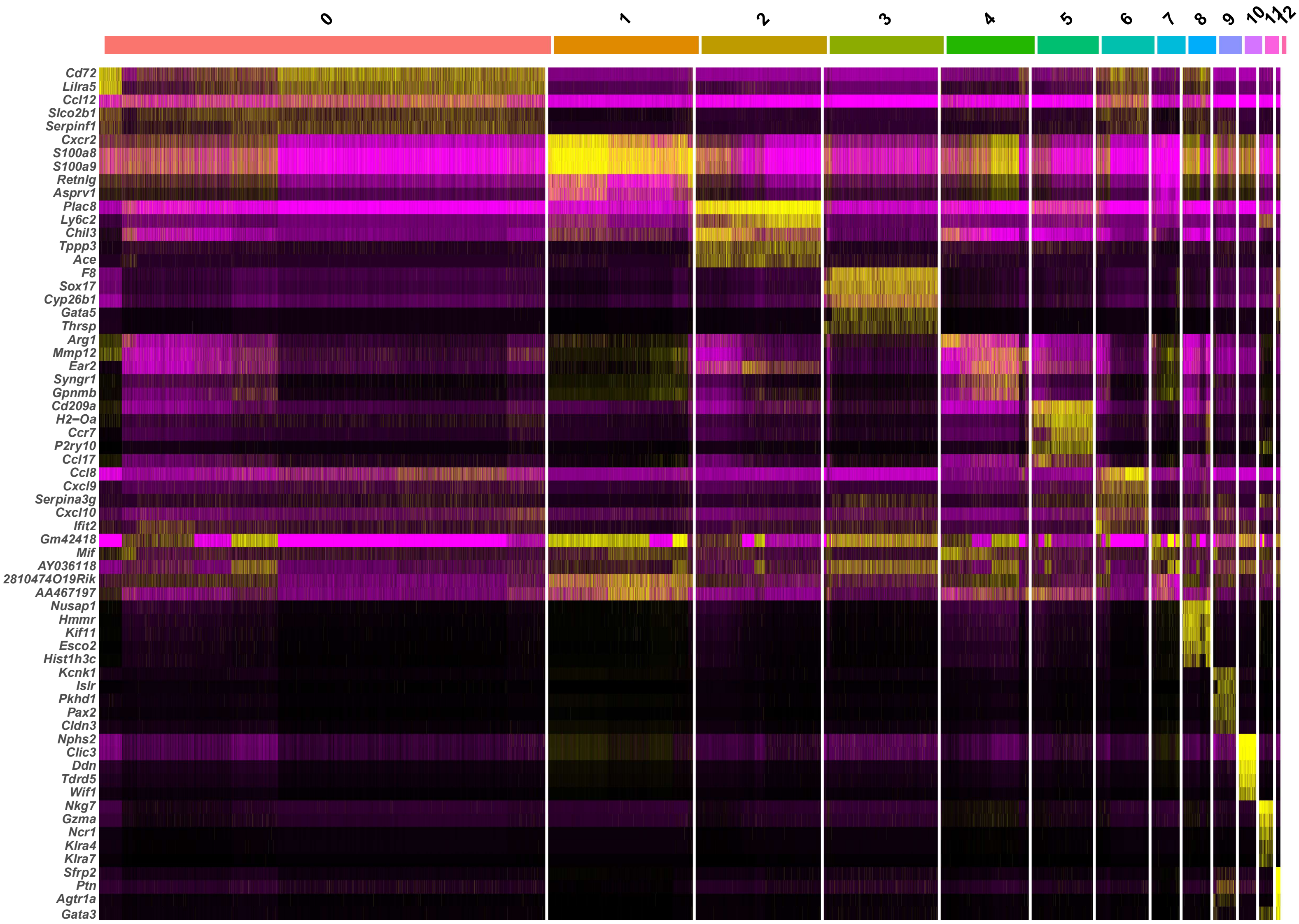


**Figure S2. Identification of MPC populations.** Heatmap showing the top five genes of each cluster from all 38,074 sorted cells. Clusters: C0, C2, C4, C5, C6, C7, C8 containing 27,544 cells were identified as mononuclear phagocytes (MPCs).

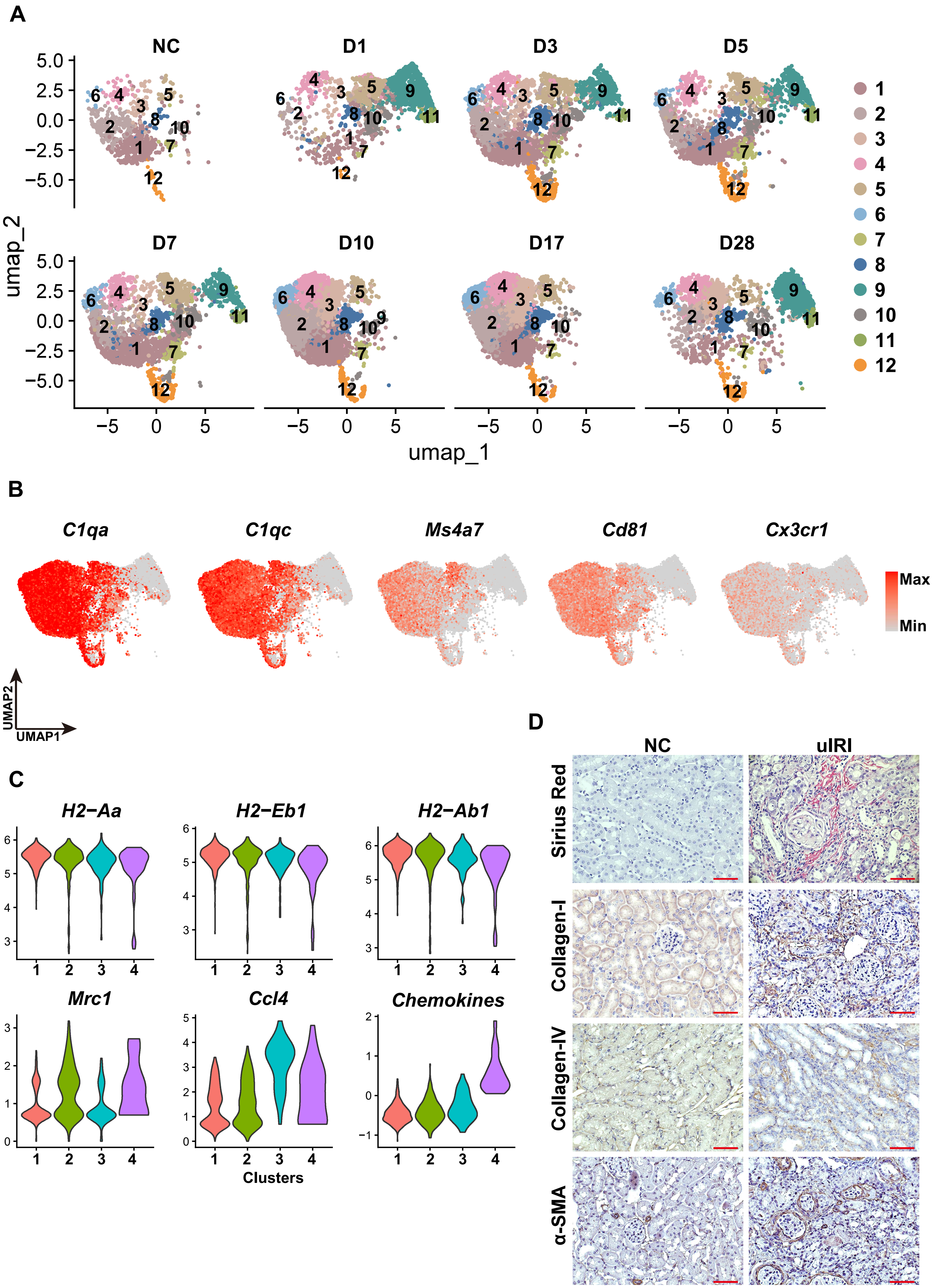

A
NC
D1
D3
D5
D7
D10
D17
D28
umap_2
umap_1
1
2
3
4
5
6
7
8
9
10
11
12
B
C1qa
C1qc
Ms4a7
Cd81
Cx3cr1
Max
Min
UMAP2
UMAP1
C
H2−Aa
H2−Eb1
H2−Ab1
Mrc1
Ccl4
Chemokines
Clusters
D
NC
uIRI
Sirius Red
Collagen-I
Collagen-IV
α-SMA

**Figure S3. Features of macrophage populations in NC and at different time points post-IRI in scRNA seq data.** (A) UMAP plot showing distribution of macrophage subclusters at each time point. (B) Feature plot showing the distribution of KRM representative genes on the UMAP. (C) Violin plot showing the representative genes of KRM cluster C1-C4 in NC (D0) kidney. “Chemokines” is a curated gene set comprising Ccl2, Ccl7, Ccl12, Cxcl2, Ccl3, and Ccl4. (D) Representative images of immunohistological staining of fibrosis markers on kidney tissues from NC and day 17 post-uIRI. Ten images were taken for each of the 17 kidney samples collected at the chronic phase (day 10, day 17, day 28) post-uIRI to quantify the percentage of fibrosis area. The data were used to perform the correlation analysis with the percentage of CD38+ macrophages in the corresponding kidney. The correlation analysis results are shown in Figure 2F.

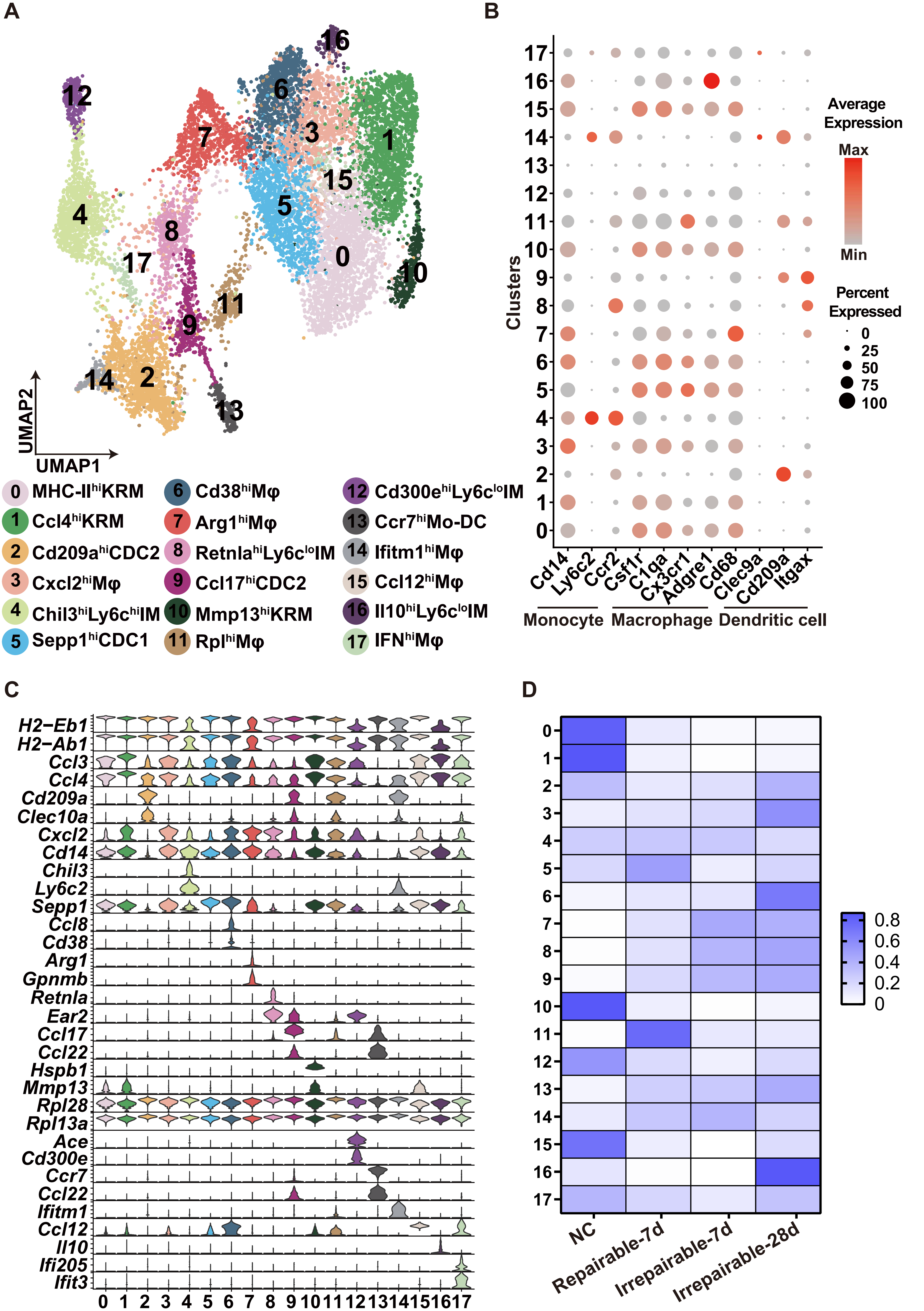

A
B
C
D
UMAP1
UMAP2
0 MHC-II^hi KRM
1 Ccl4^hi KRM
2 Cd209a^hi CDC2
3 Cxcl2^hi Mφ
4 Chil3^hi Ly6c^hi IM
5 Sepp1^hi CDC1
6 Cd38^hi Mφ
7 Arg1^hi Mφ
8 Retnla^hi Ly6c^lo IM
9 Ccl17^hi CDC2
10 Mmp13^hi KRM
11 Rpl^hi Mφ
12 Cd300e^hi Ly6c^lo IM
13 Ccr7^hi Mo-DC
14 Ifitm1^hi Mφ
15 Ccl12^hi Mφ
16 Il10^hi Ly6c^lo IM
17 IFN^hi Mφ
Clusters
Average Expression
Max
Min
Percent Expressed
0
25
50
75
100
Cd14
Ly6c2
Ccr2
Csf1r
C1qa
Cx3cr1
Adgre1
Cd68
Clec9a
Cd209a
Itgax
Monocyte
Macrophage
Dendritic cell
H2−Eb1
H2−Ab1
Ccl3
Ccl4
Cd209a
Clec10a
Cxcl2
Cd14
Chil3
Ly6c2
Sepp1
Ccl8
Cd38
Arg1
Gpnmb
Retnla
Ear2
Ccl17
Ccl22
Hspb1
Mmp13
Rpl28
Rpl13a
Ace
Cd300e
Ccr7
Ccl22
Ifitm1
Ccl12
Il10
Ifi205
Ifit3
NC
Repairable-7d
Irrepairable-7d
Irrepairable-28d
0.8
0.6
0.4
0.2
0

**Figure S4. Re-analyzing of CD38$^{hi}$ macrophages in repairable and irreparable models of IRI.** (A) UMAP plot colored by MPC clusters depicting the MPC annotation. (B) Dot plot of markers for monocyte (*Cd14, Ly6c2, Ccr2*), macrophage (*Csf1r, C1qa, Cx3cr1, Adgre1*), and dendritic cell (*Clec9a, Cd209a, Itgax*) in each MPC cluster. (C) Stacked violin plot of key genes in each MPC cluster. (D) Heatmap showing the proportion of each cluster in each indicated group and at each timepoint. The horizontal ratio of the chart adds up to 1.

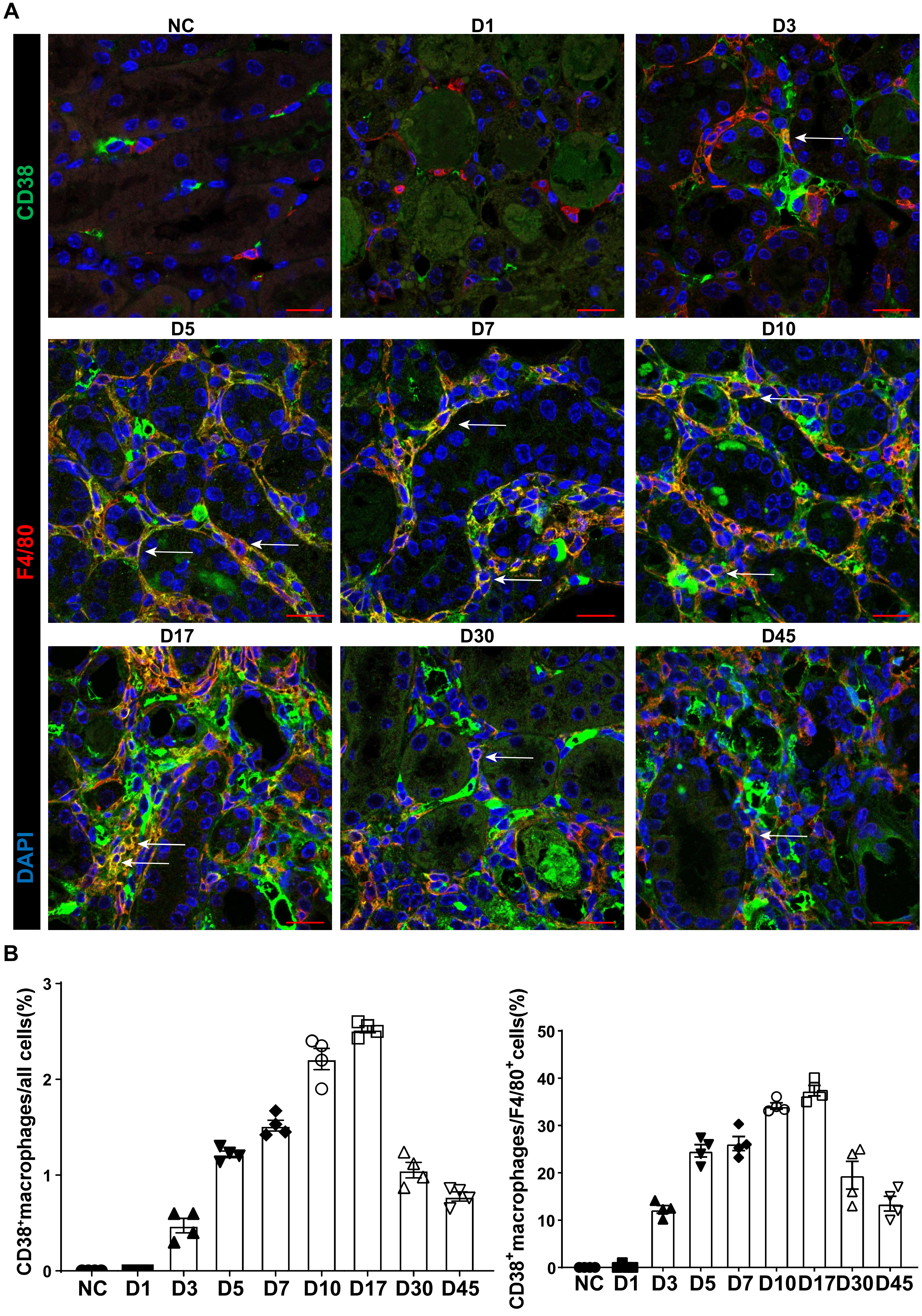

A
NC
D1
D3
D5
D7
D10
D17
D30
D45
CD38
F4/80
DAPI
B
CD38⁺macrophages/all cells(%)
0
1
2
3
NC D1 D3 D5 D7 D10 D17 D30 D45
CD38⁺macrophages/F4/80⁺cells(%)
0
10
20
30
40
50
NC D1 D3 D5 D7 D10 D17 D30 D45

**Figure S5. The distribution of CD38$^{hi}$ macrophages at different time points pot-uIRI.** (A) Representative images of immunofluorescent staining of CD38 (Green) and F4/80 (Red) in normal control or day1, day3, day5, day7, day10, day17, day30, and day45 post-injury kidney tissues. (B) Statistics of the percentage of CD38+ macrophages in all of the kidney cells and in F4/80+ macrophages at each time point. Ten images were taken for each sample. n=4 for each time point.

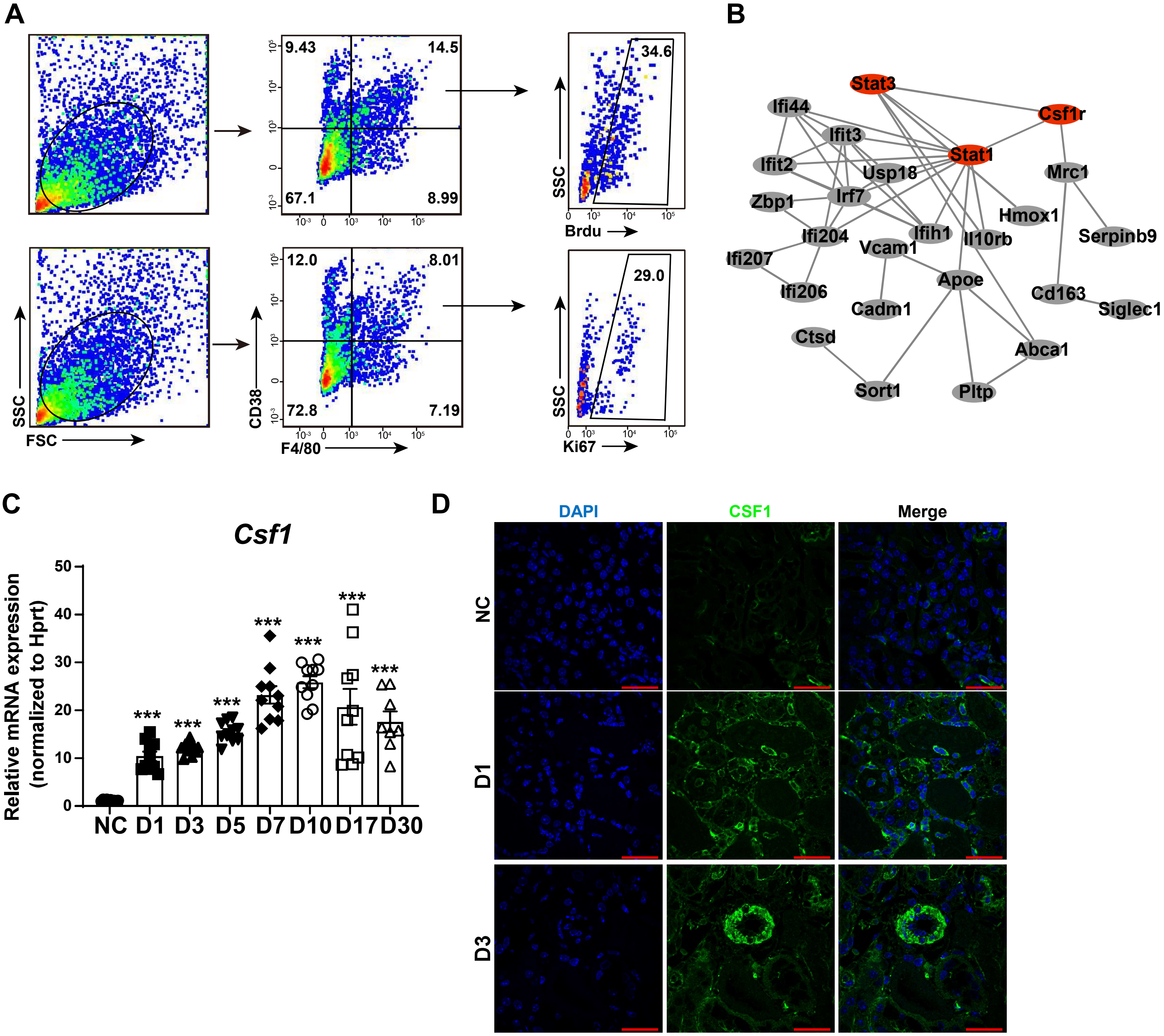


**Figure S6. Csf1 induces CD38$^{hi}$ macrophage differentiation.** (A) Flow cytometry showing proliferating CD38hi macrophage at day 7 post-uIRI. (B) Protein-protein interaction (PPI) enrichment analysis of genes from developmental module 3 in Figure 4d. (C) Relative mRNA levels of *Csf1* in the kidney at different time points post-uIRI. *** P < 0.001 compared with the normal control (NC) group, Student's t test. (D) Representative images of Csf1 immunofluorescent staining (green) in NC and kidney sections from day 1 and day 3 post-uIRI. Scale bar, 50μm.

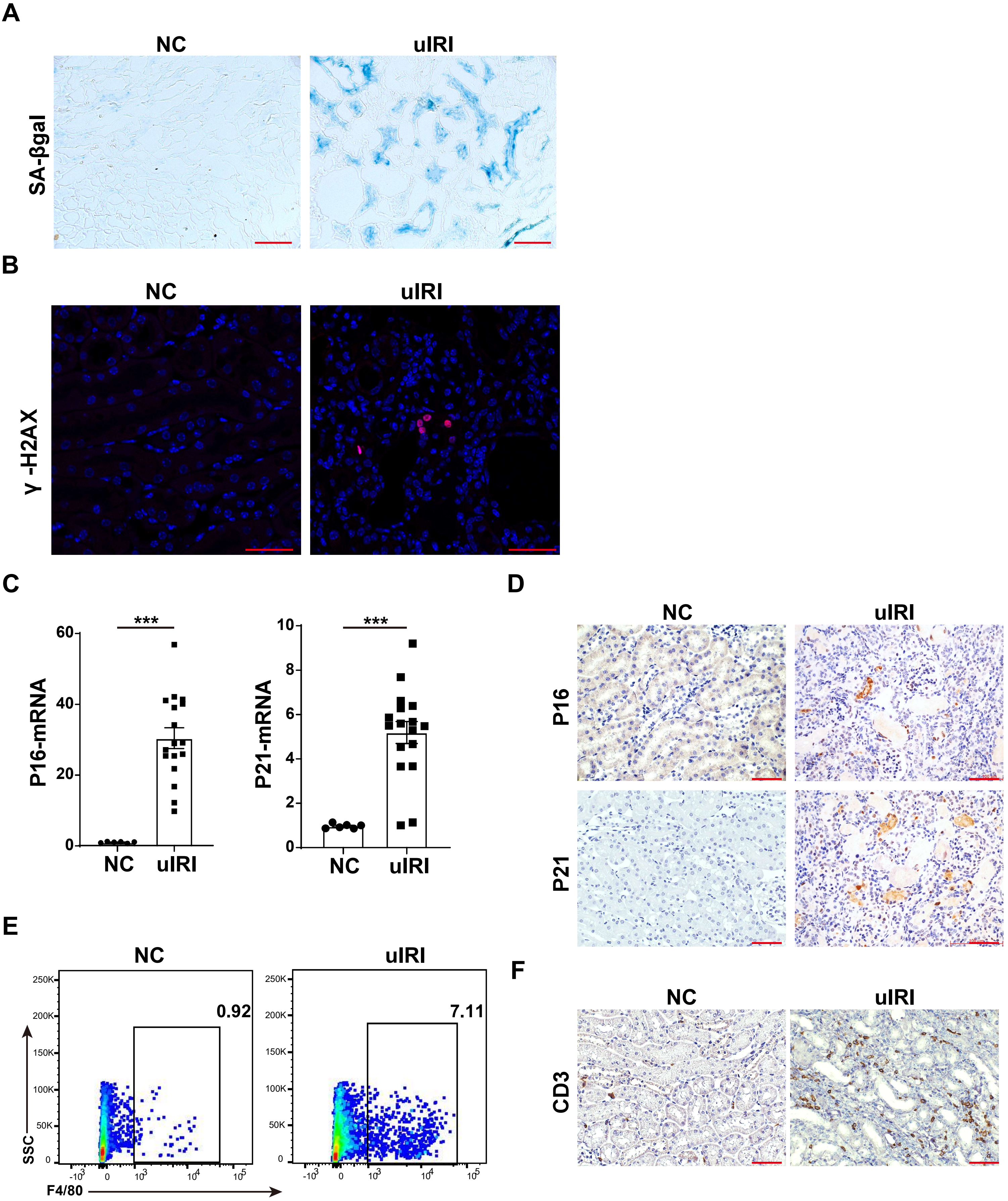


**Figure S7. Representative raw images for Figure 5C-H correlation analysis.** (A) Representative images for Figure 5C. (B) Representative images for Figure 5D. (C) Representative raw data for Figure 5E. (D) Repre-sentative images for Figure 5F. (E) Representative flow cytometry plot for Figure 5G. (F) Representative images for Figure 5H.

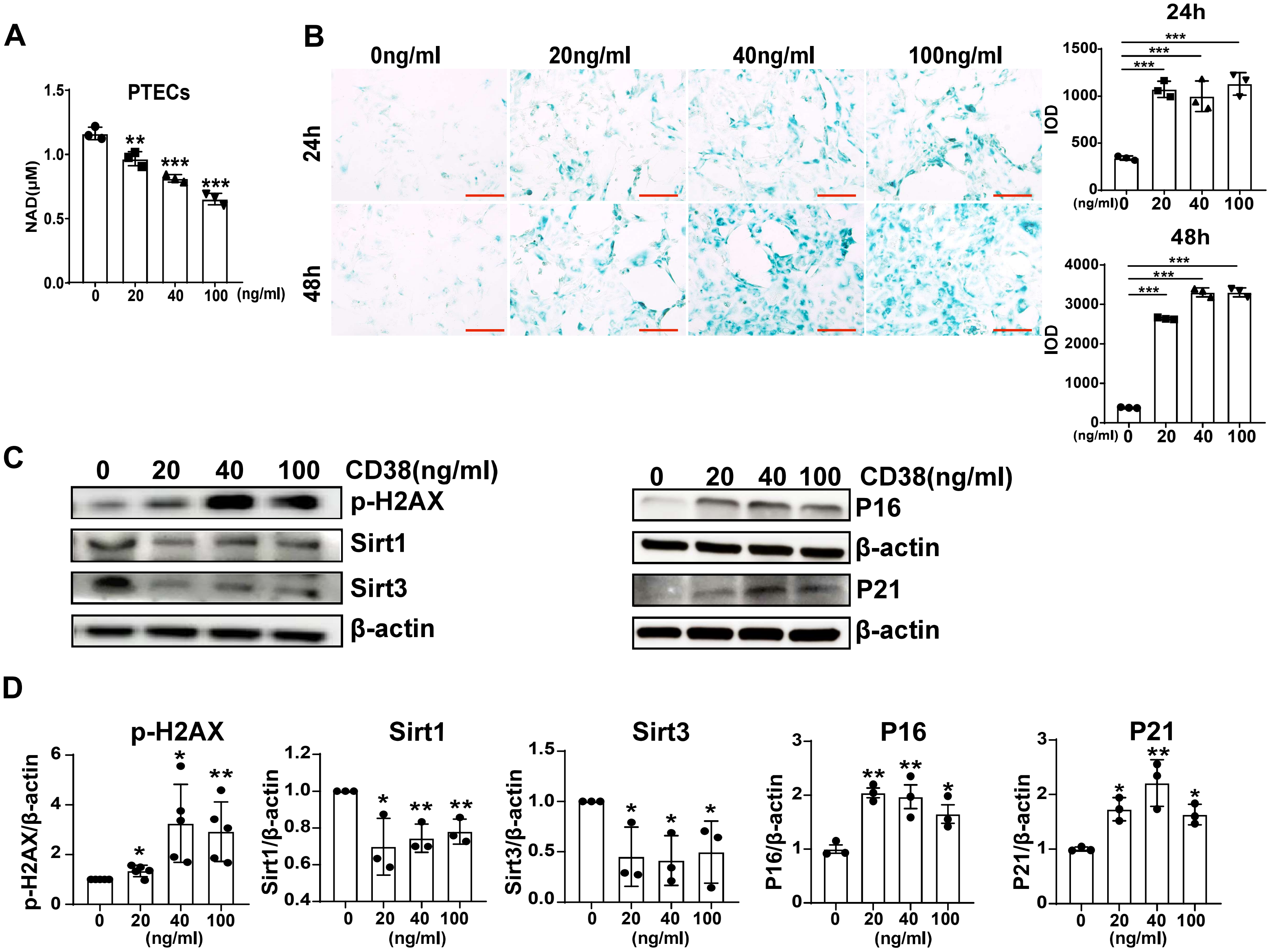


**Figure S8. CD38 promotes the senescence of PTECs.** (A) The NAD concentration in PTECs after stimulation with CD38 recombinant protein at different concentrations for 24 h. n=3. Student's t test. ** P < 0.01, *** P < 0.001. (B) Representative images of SA-β-gal staining after different doses of CD38 recombinant protein treatment to PTECs. Scale bar 70 μm. Ten images were taken and averaged SA-β-gal positive IOD was calculated for each sample. Student's t test. * P < 0.05, ** P < 0.01. (C) PTECs were treated with different doses of CD38 recombinant protein for 48 h, followed by immunoblotting with the indicated antibodies. (D) Quantification of relative protein expression level. Results are expressed as the mean ± SD. n=3. Student's t test. * P<0.05, ** P<0.01. PTEC, primary cultured tubular epithelial cells. IOD, integrated optical density.

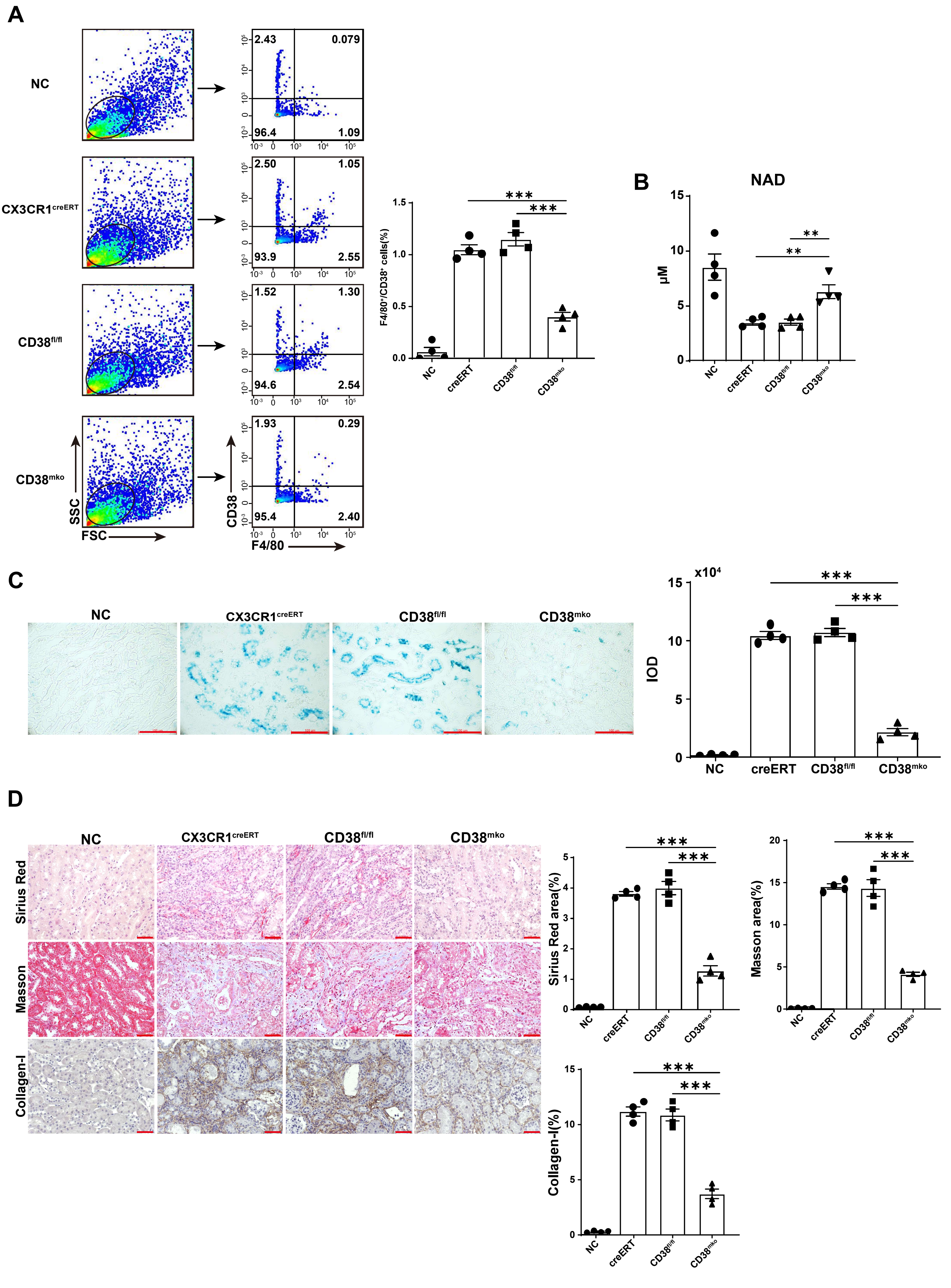
A
NC
CX3CR1creERT
CD38fl/fl
CD38mko
SSC
FSC
CD38
F4/80
2.43
0.079
96.4
1.09
2.50
1.05
93.9
2.55
1.52
1.30
94.6
2.54
1.93
0.29
95.4
2.40
F4/80+/CD38+ cells(%)
NC
creERT
CD38fl/fl
CD38mko
B
NAD
μM
C
NC
CX3CR1creERT
CD38fl/fl
CD38mko
x10⁴
IOD
NC
creERT
CD38fl/fl
CD38mko
D
NC
CX3CR1creERT
CD38fl/fl
CD38mko
Sirius Red
Masson
Collagen-I
Sirius Red area(%)
Masson area(%)
Collagen-I(%)

**Figure S9. Results of macrophage-specific knockout of Cd38 at day 27 post-uIRI.** (A) Flow cytometry plot showing the number of $CD38^{hi}$ macrophages at day 27 post-uIRI in each indicated group. n=4. Student's t test. ** P < 0.01, *** P<0.001. (B) Quantification of kidney NAD+ level at day 27 post-uIRI. Student's t test. ** P < 0.01. (C) Representative images of SA-β-gal staining of kidney sections at day 27 post-uIRI and semi quantitative analysis in each indicated group. n=4. Student's t test. ** P < 0.01. (D) Representative images of Sirius Red, Masson, and Collagen-I staining of kidney sections at day 27 post-uIRI and semi-quantitative analysis in each indicated group. n=4. Student's t test. *** P <0 .001. Sham operated right kidney as normal control (NC). All scale bar, 100 μm.

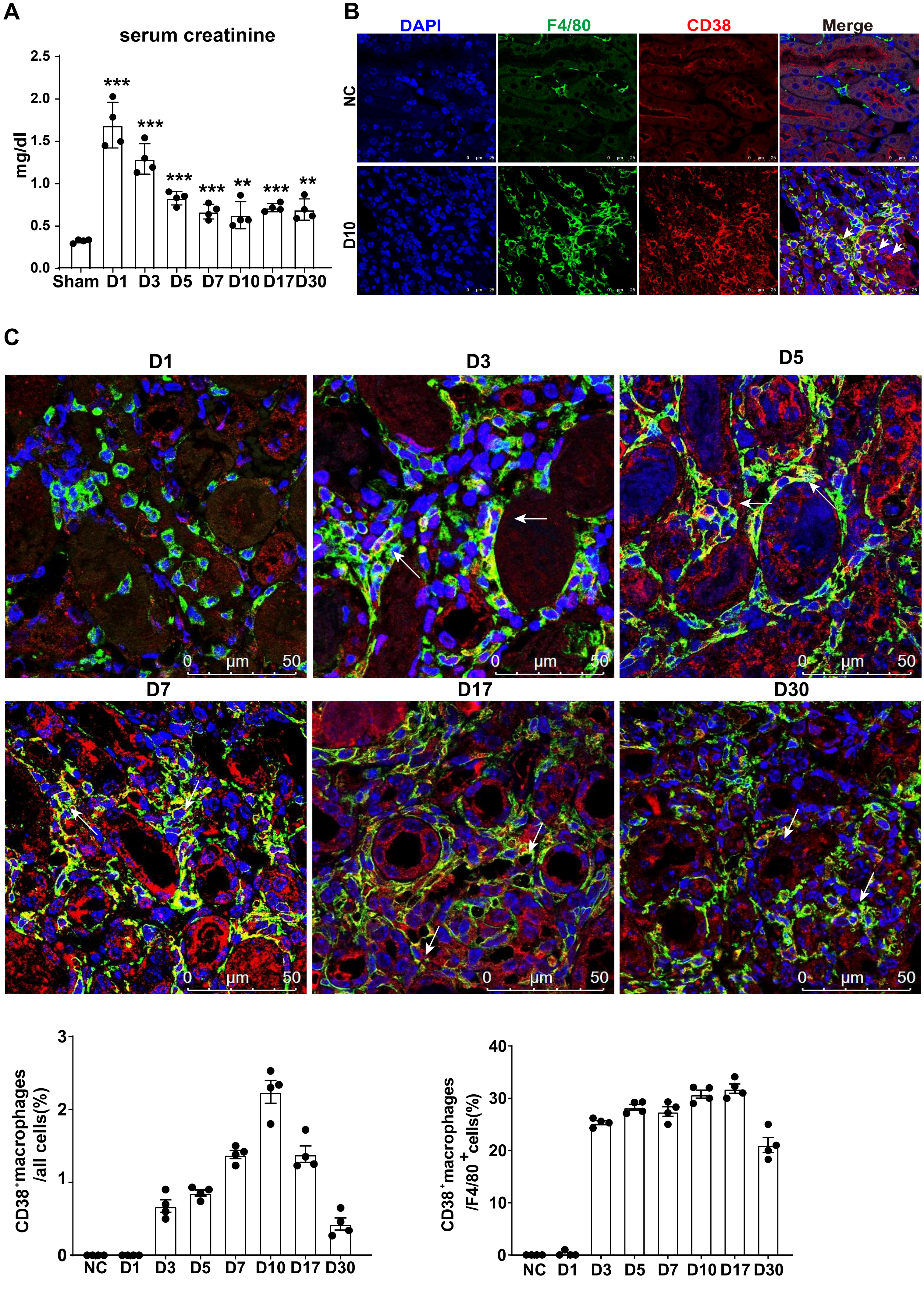

A
serum creatinine
mg/dl
2.5
2.0
1.5
1.0
0.5
0.0
***
***
***
***
**
***
**
Sham D1 D3 D5 D7 D10 D17 D30
B
DAPI
F4/80
CD38
Merge
NC
D10
C
D1
D3
D5
D7
D17
D30
0 μm 50
CD38+macrophages /all cells(%)
3
2
1
0
NC D1 D3 D5 D7 D10 D17 D30
CD38+macrophages /F4/80+cells(%)
40
30
20
10
0
NC D1 D3 D5 D7 D10 D17 D30

**Figure S10. CD38hi macrophages expression in bilateral IRI (bIRI) mouse model.** (A) The serum creatinine level in sham or at different time points post-bIRI from C57BL/6j mice. (B) Single channel and merged immunofluorescent images of F4/80 (green), CD38 (red) and DAPI (blue) in kidney tissues from NC and day 10, post-bIRI in C57BL/6j mice. (C) Representative merged immunofluorescent images of F4/80 and CD38 in kidney tissues from day1, 3, 5, 7, 17 and 30 post-bIRI in C57BL/6j mice. White arrows indicate CD38 and F4/80 double positive cells. Scale bar, 50 μm. The percentage of CD38+ macrophages in the whole kidney cells were quantified for each indicated time point.

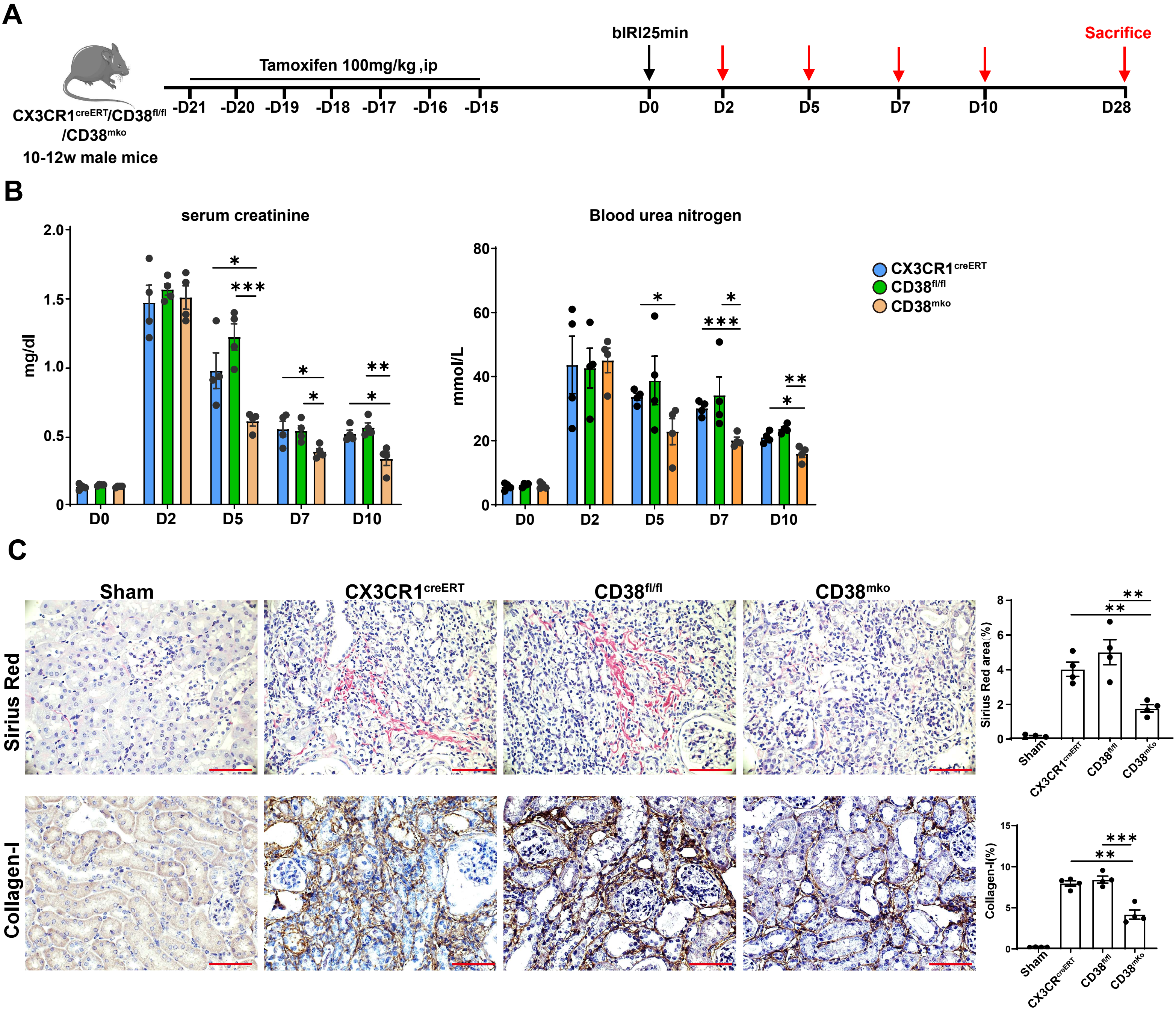


**Figure S11. Macrophage-specific knockout of Cd38 protects renal function and alleviates fibrosis progression in the bIRI model.** (A) Flow chart of tamoxifen induction protocol in bIRI animal model of CD38 control and KO mice. (B) The serum creatinine and BUN levels at different time points in bIRI mouse model in CD38 control and KO mice. n=4. Student's t test. * P<0.05, ** P<0.01, *** P<0.001. (C) Representative images of Sirius Red and Collagen-I staining of kidney sections at day 28 post-bIRI and semi-quantitative analysis in each indicated group. n=4. Student's t test. ** P < 0.01, *** P <0 .001. Scale bar, 100 μm.

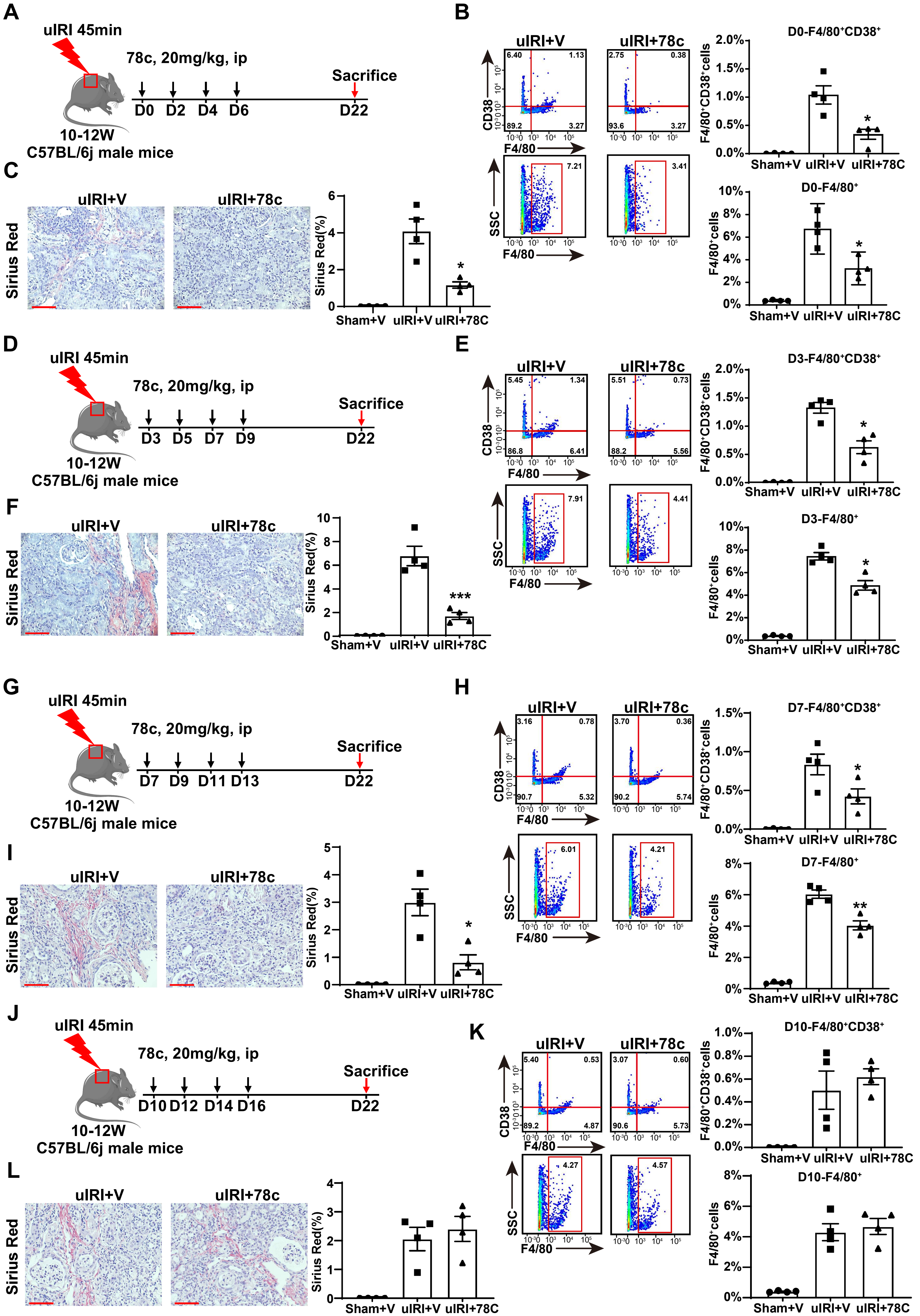
A
uIRI 45min
78c, 20mg/kg, ip
Sacrifice
D0 D2 D4 D6
D22
10-12W
C57BL/6j male mice
B
uIRI+V
uIRI+78c
D0-F4/80+CD38+
D0-F4/80+
C
Sirius Red
Sirius Red(%)
Sham+V uIRI+V uIRI+78C
D
D3 D5 D7 D9
E
D3-F4/80+CD38+
D3-F4/80+
F
G
D7 D9 D11 D13
H
D7-F4/80+CD38+
D7-F4/80+
I
J
D10 D12 D14 D16
K
D10-F4/80+CD38+
D10-F4/80+
L
CD38
F4/80
SSC
F4/80+CD38+cells
F4/80+cells

**Figure S12. The therapeutic effects of different administration strategies for 78c in the uIRI model.** (A, D, G, J) Flow charts of different drug treatment strategies in uIRI animal models. (B, E, H, K) Flow cytometry plots showing the percentage of kidney Cd38+/F4/80+ macrophages and F4/80+ macrophages at day 22 post-uIRI under different drug treatment strategies. (C, F, I, L) Representative images of Sirius Red staining of kidney sections at day 22 post-uIRI under different drug treatment strategies. n=4 for each indicated group. Student's t test. * P <0 .05, *** P <0 .001.

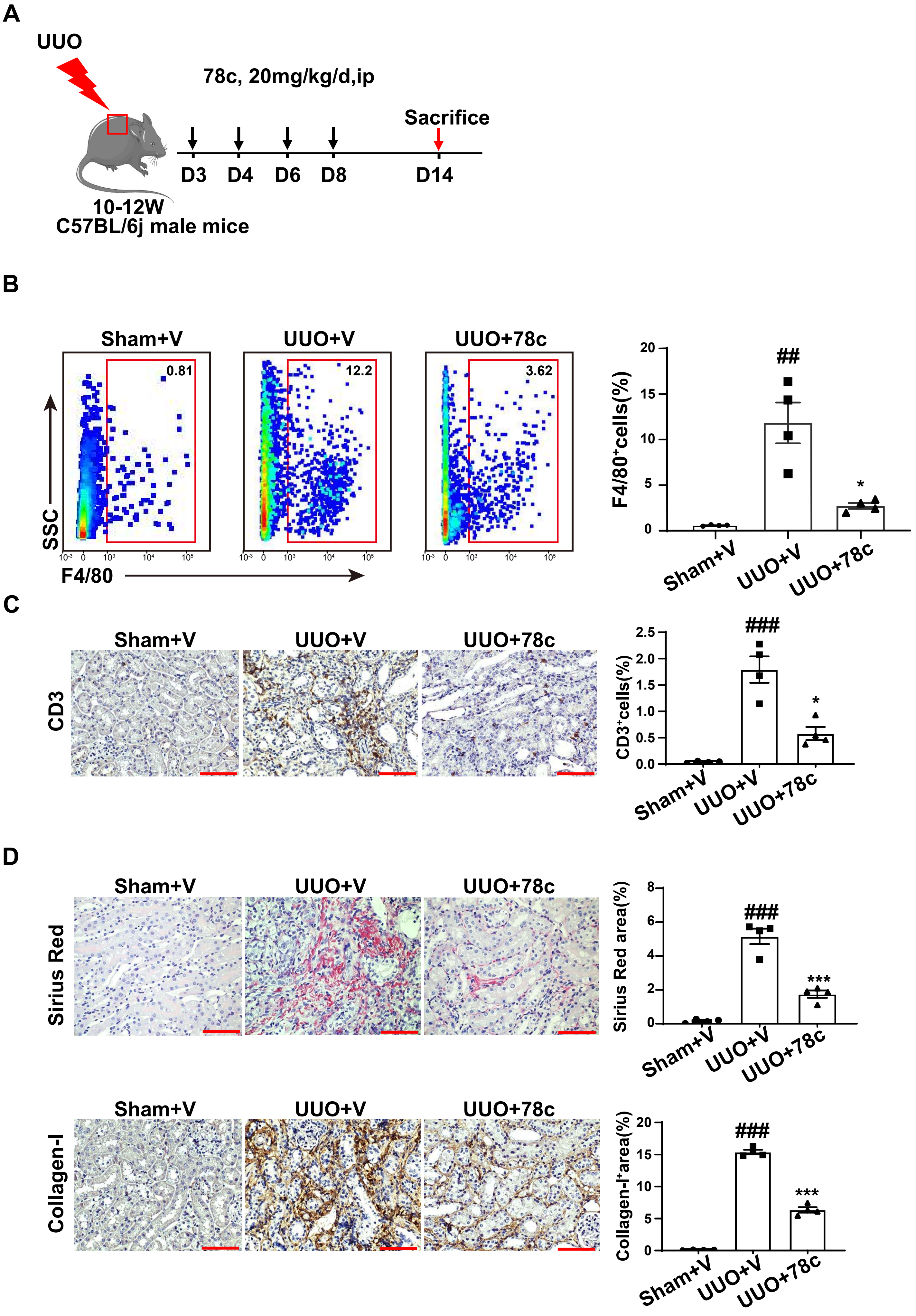

A
UUO
78c, 20mg/kg/d,ip
Sacrifice
D3
D4
D6
D8
D14
10-12W
C57BL/6j male mice
B
Sham+V
UUO+V
UUO+78c
0.81
12.2
3.62
SSC
F4/80
F4/80⁺cells(%)
##
*
C
CD3
CD3⁺cells(%)
###
*
D
Sirius Red
Sirius Red area(%)
###
***
Collagen-I
Collagen-I⁺area(%)
###
***

**Figure S13. Inhibition of CD38 by small molecular inhibitor 78c alleviates fibrosis progression in UUO model.** (A) Flow chart of drug treatment strategy in UUO animal model. (B) Flow cytometry plots showing the percentage of kidney F4/80+ macrophages and quantification result. (C) Representative images of Cd3+ T cell staining of kidney sections. (D) Representative images of Sirius Red and Collagen-I staining of kidney sections. Student's t test. # P < 0.05, ## P < 0.01, ### P < 0.001 compared to Sham+V group, * P < 0.05, ** P < 0.01, *** P < 0.001 compared to uIRI+V group. Scale bar, 50 μm. V, vehicle.

A

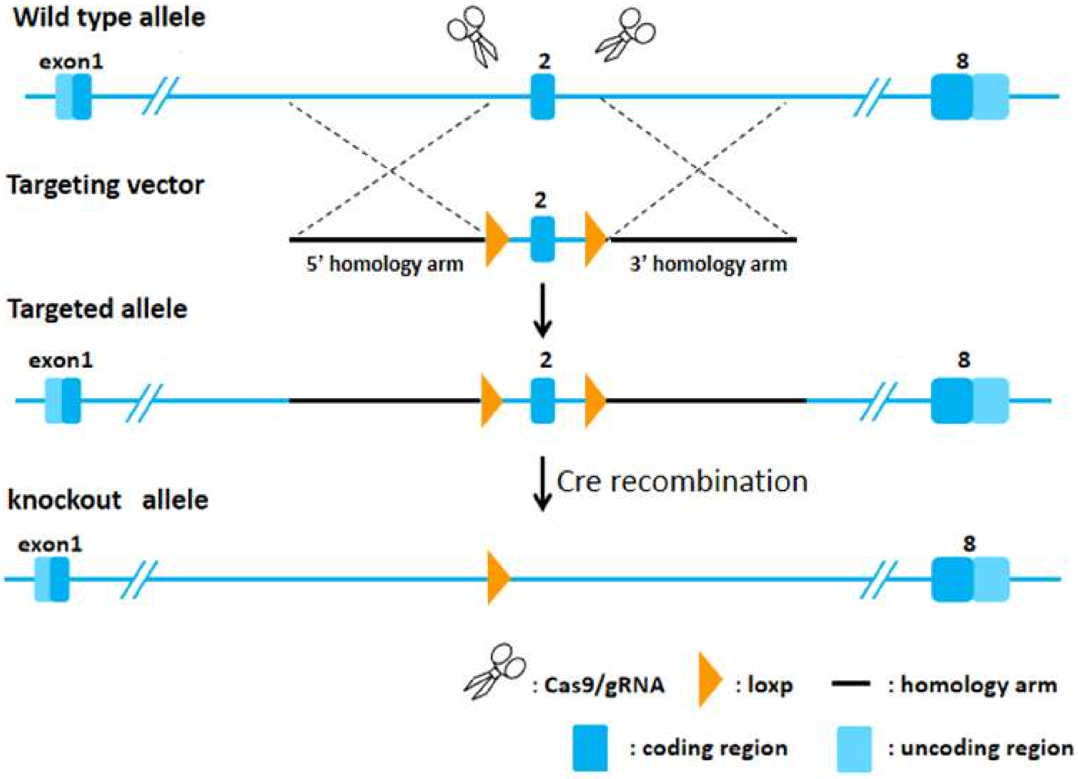


B

| gRNAs | Sequence (5'-3') |
| --- | --- |
| gRNA1 | CATCTGGAATCATATACCCAGGG |
| gRNA2 | TACCTGGTTTGCGCCTCCAGAGG |

C

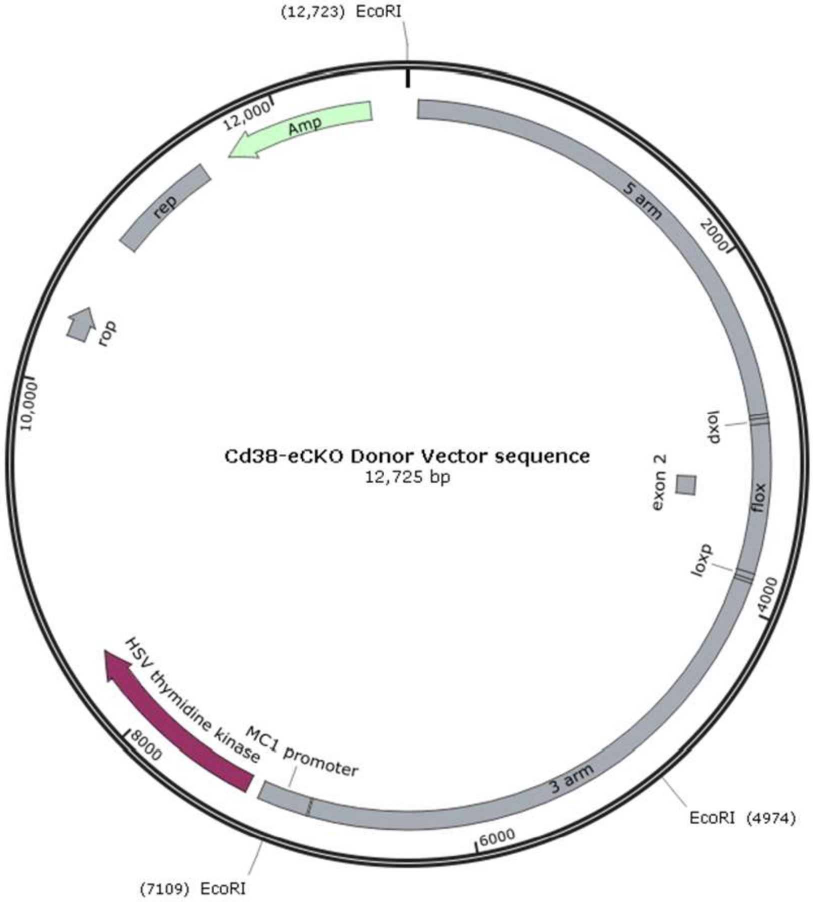

**Figure S14. The construction strategy of CD38mko mice.** (A) The schematic diagram of generation of CD38mko mice. (B) Sequence information of guide RNAs (gRNAs). (C) The construction diagram of the homologous recombination vector plasmid.

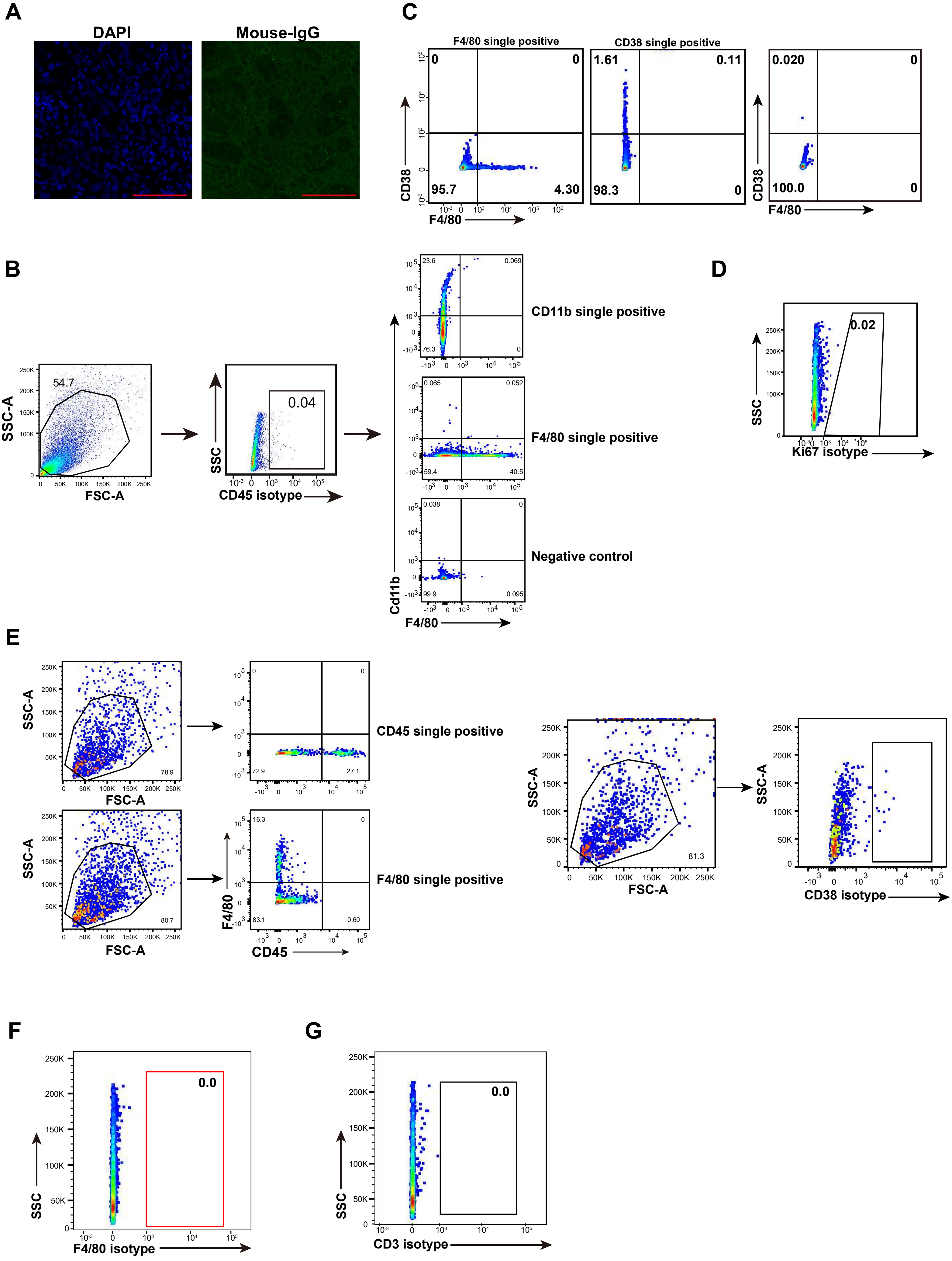
A
DAPI
Mouse-IgG
C
F4/80 single positive
CD38 single positive
0
0
95.7
4.30
1.61
0.11
98.3
0
0.020
0
100.0
0
CD38
F4/80
B
54.7
SSC-A
FSC-A
0.04
SSC
CD45 isotype
23.6
0.069
76.3
0
CD11b single positive
0.065
0.052
59.4
40.5
F4/80 single positive
0.038
0
99.9
0.095
Negative control
Cd11b
F4/80
D
0.02
SSC
Ki67 isotype
E
78.9
SSC-A
FSC-A
0
0
72.9
27.1
CD45 single positive
80.7
16.3
0
83.1
0.60
F4/80 single positive
F4/80
CD45
81.3
SSC-A
FSC-A
SSC-A
CD38 isotype
F
0.0
SSC
F4/80 isotype
G
0.0
SSC
CD3 isotype

**Figure S15. Flow cytometry experiment controls.** (A) Staining by isotype control IgG of Cd38 on kidney sections. (B) FMO control for Figure S1D. (C) FMO control for Figures 2E and 4G. (D) FMO control for Figure S6A. (E) FMO control for Figure 6H. (F) FMO control for Figure S13b. (G) FMO control for Figure 7K.

**Supplemental Tables**

**Table S1.** Top 200 DEGs in 13 clusters of all sorted cells.

Supplemental File (Excel)

**Table S2.** Top 200 DEGs in 15 clusters of MPCs.

Supplemental File (Excel)

**Table S3.** Gene sets used for functional characterization and scoring.

Supplemental File (Excel)

**Table S4**. Antibodies and materials.

Supplemental File (Excel)

**Table S5 Characteristics of patients with AKI**

| Patient No. | Etiology | Biopsy Scr (μmol/L) | Peak Scr (μmol/L) | AKI or AKD | Peak stage | ATI severity | Recovery |
|---|---|---|---|---|---|---|---|
| 1 | 1 | 352 | 591 | 1 | 3 | severe | 2 |
| 2 | 1 | 464 | 1702 | 1 | 3 | mild | 1 |
| 3 | 2 | 148 | 173 | 2 | 1 | mild | 2 |
| 4 | 2 | 177 | 365 | 1 | 3 | mild | 2 |
| 5 | 1 | 262 | 684 | 1 | 3 | severe | 2 |
| 6 | 2 | 231 | 1568 | 1 | 3 | severe | 1 |
| 7 | 1 | 324 | 423 | 3 | 3 | severe | 1 |
| 8 | 2 | 96 | 1241 | 1 | 3 | severe | 1 |
| 9 | 2 | 102.5 | 679 | 1 | 3 | mild | 1 |
| 10 | 2 | 75.8 | 84 | 3 | / | mlid | 1 |
| 11 | 2 | 113 | 162 | 1 | 1 | mild | 1 |
| 12 | 1 | 133 | 224 | 1 | 2 | mild | 2 |
| 13 | 4 | 51.7 | 69 | 2 | 1 | mild | 1 |
| 14 | 4 | 224.4 | 995 | 1 | 3 | severe | 1 |
| 15 | 2 | 303 | 777 | 1 | 3 | severe | 2 |
| 16 | 2 | 66.8 | 106 | 1 | 1 | mild | 1 |
| 17 | 1 | 149.8 | 160.1 | 2 | 1 | mild | 2 |
| 18 | 2 | 130.8 | 152 | 2 | 1 | mild | 1 |
| 19 | 1 | 189.2 | 685 | 1 | 3 | severe | 1 |
| 20 | 2 | 166.3 | 683 | 1 | 3 | mild | 1 |
| 21 | 1 | 722.5 | 1345 | 1 | 3 | severe | 2 |
| 22 | 2 | 1157 | 1239 | 1 | 3 | severe | 1 |
| 23 | 2 | 510 | 969 | 1 | 3 | severe | 2 |
| 24 | 2 | 162 | 262 | 1 | 2 | severe | 2 |
| 25 | 2 | 256 | 533 | 1 | 3 | mild | 1 |
| 26 | 2 | 184.9 | 672 | 1 | 3 | severe | 2 |
| 27 | 2 | 156 | 870 | 1 | 3 | mild | 1 |
| 28 | 2 | 468 | 480 | 1 | 3 | severe | 3 |

| | | | | | | | |
|---|---|---|---|---|---|---|---|
| 29 | 2 | 112 | 800 | 1 | 3 | mild | 1 |
| 30 | 2 | 798 | 866 | 1 | 3 | severe | 2 |
| 31 | 2 | 983.4 | 1366 | 1 | 3 | severe | 1 |
| 32 | 2 | 500 | 1016 | 1 | 3 | severe | 2 |
| 33 | 4 | 407.4 | 444.82 | 1 | 3 | severe | 2 |
| 34 | 2 | 124.5 | 124.5 | 2 | 1 | mild | 1 |
| 35 | 2 | 372 | 913 | 1 | 3 | severe | 1 |
| 36 | 2 | 60.4 | 82 | 2 | 1 | severe | 2 |

**Note:**
**Etiology:** 1/ischemia; 2/nephrotoxic; 3/others; 4/unknown
**AKI/AKD:** AKI=1; AKD=2; Non_AKI or Non_AKD=3
**Recovery:** 1/complete recovery; 2/partial recovery; 3/not in recovery
**Abbreviations:** AKI, acute kidney injury; AKD, acute kidney diseases; ATI, acute tubular injury

**Table S6.** rt-qPCR primers.

Supplemental File (Excel)
